# Carnot Theorem Revisited: A Critical Perspective

**DOI:** 10.3390/e27040346

**Published:** 2025-03-27

**Authors:** P. D. Gujrati

**Affiliations:** 1 Department of Physics, The University of Akron, Akron, OH 44325, USA; pdg@uakron.edu; 2 School of Polymer Science and Polymer Engineering, The University of Akron, Akron, OH 44325, USA

**Keywords:** Carnot theorem, Carnot engine, engine efficiency, Clausius construction, reversible engines, irreversible engines, hypothetical Carnot engine, regenerators

## Abstract

After a brief review of Carnot’s everlasting contributions to the foundations of thermodynamics, we critically examine the consequences of the Carnot theorem, which leaves behind some lingering questions and confusion that persist even today. What is the one significant aspect of the Carnot cycle that leads to this theorem? When does the working substance play an important role for an engine and what is its correlation with the *protocol* of *operational details?* Do all reversible engines working between the same two temperatures have the same maximum efficiency of the Carnot engine as Fermi has suggested? Are all heat engines equivalent to a Carnot engine in disguise? Our new perspective allows for the clarification of these questions with a positive answer for the last question. Recognizing that Carnot eventually abandoned the caloric theory, we use a result by Carnot and simple dimensional analysis to show how the first law, the concept of entropy, and the efficiency of the Carnot engine could have been germinated by Carnot in his time. This then demonstrates that Carnot had good understanding of entropy before its invention by Clausius. We suggest that both should be credited with inventing entropy by calling it Carnot–Clausius entropy. We also clarify some fundamental misconceptions plaguing reversible regenerators and their irreversible replacement by heat exchangers in the field.

## 1. Introduction

### 1.1. Some Historical Notes

Sadi Carnot was not only a giant in but also the founder of the field of thermodynamics as his contribution [1] published in 1824 paved the way not only for the second law by Clausius [2] but also the first law that was discovered later by Joule [3] and Mayer [4] in the 1840s [5]. Erlichson [6], Coopersmith [7], and Moreau and Pomeau [8] provide a very good historical perspective about Carnot, which should be a good overview, along with Jaynes’s discussion [9] of Carnot’s technical reasoning and its generality. Some have cast doubt on the genius of Carnot by casting aspersion for his use of the by-now discredited caloric theory of heat that was prevalent in his days. Güėmez, Fiolhais, and Fiolhais [5] reproduce the original calculation of Carnot, but interpret his work by using modern data.

**Figure 1:**
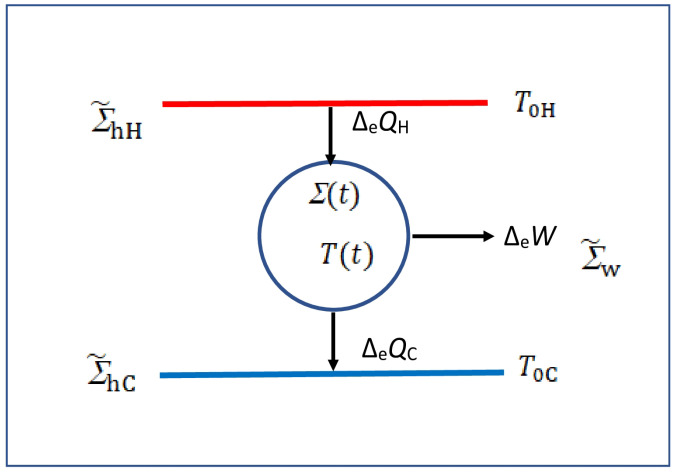
Schematic form of an irreversible heat engine $E^{\mathrm{Irr}}$ running between two heat mediums ${\tilde{\Sigma }}_{\mathrm{hH}}$ and ${\tilde{\Sigma }}_{\mathrm{hC}}$ at instantaneous temperatures $T_{0H}\left(t\right)$ and $T_{0C}\left(t\right)$, respectively; its instantaneous temperature T(t) may be different from $T_{0H}\left(t\right)$ or $T_{0C}\left(t\right)$ over the irreversible cycle of $E^{\mathrm{IrR}}$. The exchange macroheats $\Delta_{e}Q_{H},$$\Delta_{e}Q_{C}$, and $\Delta_{e}W$ are defined over the cycle so they have no *t* dependence, and ensures that the engine executes it cycle over and over again.

It is very clear from his memoir that Carnot has a deep understanding of the general nature of his result that the performance of a reversible (ideal) engine depends not on the nature of the engine but on the temperatures (always taken *non-negative* in this study) of the two heat mediums; the corresponding Carnot engine $E^{C}$, which may be reversible (denoted by $E^{\mathrm{RC}}$) or irreversible (denoted by $E^{\mathrm{IrC}}$), is shown as a generic heat engine in Figure 1. Thus, he seems to have been the first one to introduce the concept of reversibility for the performance. After the publication of *Reflections* [1], he had concluded that the hypothesis that caloric was a form of matter must be abandoned [1] (p. 219). He then put forward the hypothesis that heat was due to the motion of matter, not matter itself, and that heat and motive power are *interconvertible*. He wrote the following on p. 225:

*Reflections*-1: “Heat is simply motive power, or rather motion which has changed its form. It is a movement among the particles of bodies. Wherever there is destruction of motive power, there is at the same time production of heat in quantity exactly proportional to the quantity of motive power destroyed. Reciprocally, wherever there is destruction of heat, there is production of motive power.”

It is clear that Carnot was aware that heat and mechanical work were interconvertible, which is basically an ingredient in the foundations of the science of thermodynamics. It is commonly stated that Carnot was not familiar with the principle of energy conservation, but the quote above shows that this is not the case. Kuhn [10] (see footnote 1 there) highlights the “presumptive evidence” for energy conservation by Carnot, which he was able to rationalize by computation carried out for an adiabatic expansion experiment (so *no* heat transfer) that Gay-Lussac had performed in 1807. So, the heat Carnot is referring to is generated internally. We will come back to this point later; see Section 3, where we derive the first law in Equation (21) by using arguments available to Carnot at his time.

There has been serious concern that Carnot’s writing has been misinterpreted, which has made assessing his contributions to the formulation of thermodynamics very confusing; see, for example [11,12,13,14]. In fact, La Mer [12,13] has argued that by *reinterpreting* various terms like “feu”, “calorique”, and “chaleur”, that are normally understood as synonyms for “heat” in translations such as by Thurston [1] and others as “flame”, “entropy”, and “heat”, respectively, one finds a consistency in Carnot’s writing that has been very confusing. He claims that interpreting “calorique” as “entropy” removes some misconceptions in the literature. We will also come back to this issue later, but must mention here from the outset that as Carnot had eventually rejected the caloric theory, as mentioned above, any attempt to reinterpret “calorique” as “entropy” is a *futile* exercise; however, see Section 3.4. At present, there are other more significant issues that we wish to focus on.

Most mechanical power during Carnot’s time was generated by sheer physical strength (humans and animals) or by waterwheels. While Carnot talks about waterfalls and their motive power, the intent was to think of caloric as a matter like water that falls from a hot body to a cold body, but this does not seem to be the right analogy. The motive power in waterfalls is due to gravity and the drop in height through which water falls is due to gravitational potential, and not water itself. The fall is *spontaneous* and can be stopped by creating an obstacle in its path of fall. The caloric theory is basically a theory of *calorimetry*, in which the amount of caloric (heat) transfer is due to temperature difference, but there is no loss of caloric. According to Carnot, the motive power is due to the caloric transfer from hot bodies to cold bodies as if it is a spontaneous exchange as is commonly believed. But the latter occurs only when the two bodies are in thermal contact, which never happens in E; indeed, it happens due to adiabatic connections between the two bodies so it is most certainly not spontaneous, but forced. Following the waterfall imagery, the “potential energy” change or the motive power in the caloric transfer must be given by an expression proportional to

(1) $$Q(T_{0H}-T_{0C})$$

by thinking of *Q* as “mass.” Lazare Carnot [15], the father of Sadi Carnot, was himself a brilliant person who studied math and military engineering and wrote about a perfect waterwheel, of which its energy would not be wasted or dissipated. He also mentioned that such a perfect waterwheel could run backward successfully. It is where Sadi Carnot obtained his inspiration for the heat engine.

It is, indeed, surprising that Carnot did not take the bold step to couple this calorimetric fact with the interconvertibility aspect of heat. Fortunately, as we will argue here, this oversight not only did not affect his main conclusion in his *Reflections* [1], which is now known as Carnot’s theorem, but also that his arguments can be easily extended as we do here to see the inception of the first and second laws. All of this attests to Carnot’s sheer brilliance that jumps out of the short book he published in 1824, knowing well that his computations were crude, so his reasoning was full of awe. In his own words, the theorem, which is a *cornerstone of classical thermodynamics*, has many parts, as follows:

Carnot Theorem (C-Th)

1. **C-Th-1**: Wherever there exists a difference of temperature, motive power can be produced [1] (p. 51).
2. **C-Th-2**: The maximum of motive power resulting from the employment of steam is also the maximum of motive power realizable by *any means whatever* [1] (p. 55).
3. **C-Th-3**: The motive power of heat is *independent of the agents* employed to realize it; its quantity is fixed solely by the temperature of the bodies *between which is effected*, finally, the transfer of the caloric [1] (p. 68).

The phrases italicized in C-Th-2 and C-Th-3 for emphasisare not by Carnot. We have emphasized them, as we will try to critically understand their significance. In particular, we ask the following:

Q1. Does by “*any means whatever*” mean any operational procedure, i.e., *protocol* different from those of the Carnot cycle but resulting in a reversible engine?
Q2. Does *independent of the agents* have anything to do with the interplay between the working substance (WS) and the protocol of the cycle?
Q3. Does “*between which is effected*” allow for a *collection of mediums* with temperatures lying between a chosen hot and cold temperatures $T_{0H}$ and $T_{0C}$ in Figure 1 but still result in a reversible engine?

We should be grateful to Carnot, a persistent note maker, for recording his thinking about various topics in the form of notes that have survived. They provide a much better picture of a thinker with deep understanding and outstanding generality of conclusions.

For the benefit of readers, we collect various acronyms used in the text in a table at the end.

### 1.2. Motivation

There are two parts in this contribution. The first one is an introductory part to highlight the understanding of heat engines at the time of Carnot, and what limitations were present that are no more. This part is to highlight the contribution of Carnot, a giant in the field of heat engines and a forceful founder of thermodynamics, and to acquaint the reader with his deep insight and clever thinking to help formulate principles that govern entire thermodynamics today. While I am not an expert in the history of classical thermodynamics, the belief is that this contribution to celebrate the “200 Years Anniversary of” Sadi Carnot’s *Reflections* brings out a different perspective based on my own expertise in nonequilibrium thermodynamics. As a result, I am able to interweave a *conjectural platform* to show how the law of energy conservation (the first law) could have been established by Carnot with the knowledge he possessed during his life and simple dimensional analysis. I should emphasize that the knowledge I am talking about does not emerge from his short note [1], which was meant for a general audience and not as a technical resource. For example, he abandoned the caloric theory and recognized mechanical equivalence after the publication of *Reflections*, in which I take the liberty of setting out my version of the first law. I also demonstrate that Carnot’s writings have the proper germination of the concept of entropy that was later used by Clausius [2]. Because of this,

I propose to identify this thermodynamic entropy by the *Carnot–Clausius entropy* to give him the full credit that he deserves.

The second part is more technical in nature. There is a strong focus here to carefully and critically investigate the conditions required for the validity of various parts of the Carnot theorem, to seek if and how they can be extended to other reversible engines that operationally differ from $E^{\mathrm{RC}}$ in their protocols, and the *interplay* between the reversible engine efficiency and WS. This interplay does not seem to have attracted any serious interest and critical investigation for reversible engines to the best of my knowledge, even though it is widely believed that WS plays a major role in irreversible engines. We will establish that WS is also important for reversible engines because of the above interplay.

By following Carnot’s edict to have two isotherms at different temperatures, we find it convenient to break *any* cycle $P_{\mathrm{cyc}}$ into two *disjointed* parts, only $P_{\mathrm{acc}}$ and $P_{\mathrm{rej}}$, during which exchange of macroheat $Q_{\mathrm{acc}}>0$ is accepted from and $Q_{\mathrm{rej}}<0$ rejected to the appropriate heat mediums, respectively.The cycle $P_{\mathrm{cyc}}$ may be executed reversibly or irreversibly so the partition is general.

**Definition** **1.** *We say that $P_{\mathrm{acc}}$ corresponding to $Q_{\mathrm{acc}}>0$ and $P_{\mathrm{rej}}$ corresponding to $Q_{\mathrm{rej}}<0$ refer to* hot temperatures *and* cold temperatures*, respectively, where $P_{\mathrm{cyc}}=P_{\mathrm{acc}}\cup P_{\mathrm{rej}}$.*

For example, $P_{\mathrm{acc}}$ and $P_{\mathrm{rej}}$ for $E^{C}$ include adiabats as part of them even though they do not allow any exchange of macroheats. Thus, even the Carnot cycle of $E^{C}$ can be thought of as being partitioned into exactly $P_{\mathrm{acc}}$ and $P_{\mathrm{rej}}$. It also includes the possibility in which $P_{\mathrm{acc}}$ and $P_{\mathrm{rej}}$ are directly connected to each other at their common endpoints to form $P_{\mathrm{cyc}}$. Usually, a cycle consists of many different segments, not just the two discussed above. In this case, one particular segment $P_{1}\subseteq P_{\mathrm{acc}}$ corresponding to the heat medium at the highest temperature or mediums at higher temperatures can be identified as special in some sense, so it may be useful to make a distinction between $P_{1}$ and $P_{\mathrm{acc}}$, and the accepted macroheat $Q_{\mathrm{acc}1}$ and $Q_{\mathrm{acc}}$, respectively.

**Claim** **1.** *The division of $P_{\mathrm{cyc}}$ into only two parts $P_{\mathrm{acc}}$ and $P_{\mathrm{rej}}$ strongly suggests that any reversible engine $E^{R}$ can be thought of as a reversible Carnot engine $E^{\mathrm{RC}}$ working between two hot and cold temperatures that turn out to be sort of effective temperatures introduced later.*

This will provide an answer to the fourth question in the Abstract. How far can this analogy be taken? If conducted successfully, it will be a new result, which may have some interesting and useful consequences. This is one of our main goals. It may be possible that its irreversible analog $E^{\mathrm{Ir}}$ may have some similarity with the irreversible Carnot engine $E^{\mathrm{IrC}}$. However, we will see in Section 9.2 that it is not *always* possible to find a reversible engine for an irreversible engine without *changing* the protocol. 

A cycle is usually drawn as a PV diagram *clockwise* so the macrowork *W* performed by the engine is always positive. The thermodynamic efficiency of any engine as introduced by Carnot is always defined by

(2) $$\epsilon ≐W/Q_{\mathrm{acc}}>0$$

as a positive quantity. We will identify this as the *Carnot efficiency*. The physics behind this measure is to find out the amount of exchange macrowork *W* for a given macroheat $Q_{\mathrm{acc}}$ accepted by the engine during $P_{\mathrm{acc}}$. Although $Q_{\mathrm{rej}}$ does not appear in ϵ, it determines *W*, so it also determines ϵ. In contrast, by replacing the part $P_{\mathrm{acc}}$ by a segment $P_{\mathrm{acc}}^{\prime }\subset P_{\mathrm{acc}}$ so that we replace

(3) $$Q_{\mathrm{acc}}\;\mathrm{with}\;Q_{\mathrm{acc}}^{\prime }$$

accepted along $P_{\mathrm{acc}}^{\prime }$ results in a *non-Carnot efficiency* $\epsilon_{\mathrm{NC}}$ given by

(4) $$\epsilon_{\mathrm{NC}}≐\frac{W}{Q_{\mathrm{acc}}^{\prime }}\equiv \frac{Q_{\mathrm{acc}}}{Q_{\mathrm{acc}}^{\prime }}\epsilon \geq \epsilon .$$

This efficiency is sometimes used in reversible regenerators [16] (Equation (10.57a)); see also [17,18] (for example). We will use it in Section 9 with heat exchangers, where we also discover its limitations, as seen from Conclusion 7, which justifies not taking it seriously, as it does not measure the true motive power obtained by the entire macroheat accepted by the heat engine and possible irreversibility. However, the most serious drawback of $\epsilon_{\mathrm{NC}}$ is that it is not applicable to all WS’s, which makes its use not meaningful for the Carnot theorem. In this sense, we agree with the following comment by Salter [18], where the non-Carnot efficiency has also been discussed:

“… the efficiency of the Stirling cycle with a reversible regenerator became a source of confusion, and someone tried to remove the confusion with a careless definition of heat input. Together these two mutually supportive ideas spread like a virus throughout many articles and textbooks on thermodynamics. They spread because they sell an attractive idea: if all reversible engines are alike, then Nature is simple and parsimonious….”

Therefore, we will mostly use Carnot efficiency in this study.

As we will discover, Carnot efficiency and its interplay with WS are two different issues. While ϵ is defined for any general engine $E^{R}$ and $E^{\mathrm{Ir}}$ that are run according to their prescribed protocols describing various steps during the cycle of E, its value must surely be determined by its working substance and the protocol. Because of the cycle nature, we discover that the entropy changes ΔS during $P_{\mathrm{acc}}$ and $P_{\mathrm{rej}}$ cancel out, as follows:

(5) $$\Delta S_{\mathrm{acc}}+\Delta S_{\mathrm{rej}}\equiv 0,$$

which is valid for any engine, reversible or not, for any working substance, even though each change is determined by the nature of WS. As *W* and $Q_{\mathrm{acc}}$ are also determined by the nature of WS, how come ϵ for some engines, in particular, $E^{\mathrm{RC}}$, does not depend on the WS? What conditions must be met for each situation? This is another important part of our motivation.

Various thermodynamic quantities in Figure 1 may carry an explicit time dependence during different parts of the protocol required for $E^{C}$; they may also be required to either describe irreversible processes or to denote time-varying fields of various mediums. What is impressive is that Carnot carefully distinguishes between macroheat exchange $d_{e}Q$ and macrowork exchange $d_{e}W$ so much so that the first one occurs at two different temperatures (see C-Th-1) reversibly, but the connections between the two temperatures occur reversibly (R) without any macroheat exchange with any heat medium. He claims [1] (pp. 56–57) that

*Reflections*-2: “… all change of temperature which is not due to a change of volume of the bodies can be only a useless reestablishment of equilibrium in the caloric. The necessary condition of the maximum (macrowork) is, then, *that in the bodies employed to realize the motive power of heat there should not occur any change in temperature which may not be due to a change in volume*. Reciprocally, every time that this condition is fulfilled the maximum will be attained. This principle should never be lost sight of in the construction of heat engines; it is its fundamental basis. If it cannot be strictly observed, it should at least be departed from as little as possible.”

Thus, all changes in volume must occur reversibly so that any temperature change must be due to volume changes only and not due to macroheat exchanges. Carnot imposes a very *stringent* condition on his protocol for the Carnot cycle that no temperature changes occur due to heat exchanges. This allows for isothermal (fixed temperature) macroheat exchange and volume-changing adiabatic operations with no macroheat exchange. We will relax this requirement and allow for the situation in which there is a continuous distribution of heat mediums of temperatures $T_{0}\left(t\right)$ as a function of volume or time for later use to *maintain reversible macroheat exchange*. This situation is very common in heat engines such as Stirling engine, Otto engine, and more, where regenerators play an important role, but where there are major misconceptions and misunderstandings that one finds in the literature. Therefore, we will try to get a handle on regenerators, where a non-Carnot (NC) efficiency $\epsilon_{\mathrm{NC}}>\epsilon$ is often used. We will also consider other reversible cycles in which the hot or cold isotherm is broken into two or more hot and cold isotherms, respectively, or temperature changes requiring heat exchanges at many different temperatures so that we can answer Q1–Q3 posed above.

Traditionally, the two heat mediums are taken to be at *fixed* temperatures $T_{0H}$ and $T_{0C}$ in the Carnot cycle per Carnot’s requirement. But what will happen if we consider heat mediums with varying (non-fixed) temperatures $T_{0H}\left(t\right)$ and $T_{0C}\left(t\right)$ or consider more than one hot and cold isotherms to construct reversible heat engines? Under these conditions, there is no single Carnot engine to compare their efficiencies. Does C-Th survive in its entirety? Does C-Th-2 apply to any arbitrary reversible engine and its irreversible (Ir) counterpart, not just $E^{\mathrm{RC}}$ and $E^{\mathrm{IrC}}$, which is how it is understood. Does C-Th-3 apply to these modified reversible engines? What is the interplay between WS and the cycle protocol?

We discover that there are strong reasons to introduce two distinct protocols for running engines in a cyclic process, as their consequences are very different: *Protocol A* requires a *fix sets of external heat and work mediums* that *control* the changing macrostates of WS in the heat engine during the cycle, and *Protocol B* requires a *fix form of cycle* during which WS *controls* the sets of external heat and work mediums that are continuously changing (not fixed) for the heat engine.

The above division of the protocols allows us to understand, for the first time, that the efficiency of a reversible engine is independent of WS (Protocol A) and dependent on the WS (Protocol B).

Our conclusions are the following.

**1.**  C-Th-2 applies to any arbitrary reversible engine and its irreversible counterpart *but with a prescribed protocol specifying the set of processes (operations)* and the *working substance*.
**2.**  C-Th-2 applies to reversible Carnot engine $E^{\mathrm{RC},B}$ and irreversible Carnot engine $E^{\mathrm{IrC},B}$ for the non-fixed (B) protocols of $T_{0H}\left(t\right)$ and $T_{0C}\left(t\right)$ and *prescribed working substance*.
**3.**  C-Th-3 applies to all reversible engines and not just $E^{\mathrm{RC}}$, provided they are formed under Fix-Protocols, but not to any other reversible engines formed under NonFix-Protocols.

The need for modifying C-Th-2 and C-Th-3 has already been discussed in the literature [17,18] (for example) as the original version is too broad and exceptions exist. During our investigation, we discovered that operational protocols and working substances play a very important role in extending C-Th to any reversible heat engine that differs from $E^{\mathrm{RC}}$.

### 1.3. Layout

We wish to emphasize at the outset that we employ modern notation and terminology that are not yet common in applied thermodynamics. For example, we use macrowork and macroheat instead of thermodynamic work and heat. This allows us to make contact with some of our previous results. We also use cycle and engine synonymously, except that the engine also requires specifying WS. For simplicity, we will specify a cycle as reversible or irreversible, even if not the cycle but its execution is reversible or irreversible, and allows the engine to identify it as reversible or irreversible. However, most of this study is limited to only reversible engines, except in Section 9.2, where we face irreversibility introduced by heat exchangers. Here, we also recognize the unusual possibility of cycles that always remain irreversible in that there is no reversible analog if we wish to remain dedicated to the Carnot theorem; see Conclusions 5 and 6.

In the next section, we introduce some preliminary topics that will be useful for later discussion. We begin with the nature of temperature used by Carnot, and the absolute scale that we use so that it has the dimension of energy. We also use macrowork *W* and macroheat *Q* as having the dimension of energy for reversible (using R to denote them) and irreversible engines (using Ir to denote them). Carnot’s engine and its full specification of protocol are given in Section 2.2 and Section 2.3. They are followed by the description of the two specific protocols that govern all kinds of heat engines in Section 2.4. We supplement the discussion by specifying the characteristics of any cycle in Section 2.5. The next Section 3 is one of the most important sections. We first discuss how Carnot estimated the efficiency $E^{\mathrm{RC}}$ without knowing the first law and using the now-abandoned caloric theory in Section 3.1. We then move to reporting our conjectural platform, where we derive the first law in Section 3.2, and the seed for the entropy identification in Section 3.3 that is later used by Clausius. It is here that we also obtain the mathematical expression of the efficiency $\epsilon^{\mathrm{RC}}$ of $E^{\mathrm{RC}}$ in Equation (23), which Carnot never reported. We briefly discuss some issues with the caloric theory and the false identification of calorique with entropy in Section 3.4. We discuss other reversible engines in Section 4. They include the reversible non-Carnot cycle (RNC) and associated engine $E^{\mathrm{RNC}}$ in Section 4.1 and the arbitrary reversible cycle (ARC) and associated engine $E^{\mathrm{ARC}}$ formed by the Clausius construction in Section 4.2 that both follow NonFix-Protocol B. We conclude that the efficiency is WS-dependent for both cycles. The formation of $E^{\mathrm{ARC}}$ justifies considering it as a modification of $E^{\mathrm{RC}}$ with its two isotherms replaced by a *continuum of heat mediums* over a finite range, just as in the functioning of $E^{\mathrm{RNC}}$. It is this continuum of heat mediums that qualifies the protocol to be NonFix-Protocol B. We contrast the cycle profile of $E^{\mathrm{ARC}}$ with that of the cycle formed by two reversible Carnot engines *connected together in parallel* in Section 5, and then generalize the result to having any finite number ν of reversible Carnot engines connected together in parallel. The finite set of fixed isotherms qualifies the protocol to be Fix-Protocol A. This comparison is made to justify Equations (8c) and (8d).

We verify the conclusions of Section 3 à la Carnot by considering $E^{\mathrm{RC}}$ in its complete detail in Section 6 using post-Carnot thermodynamics, where we have the recourse to use the first and the second laws. We use the first law to determine the efficiency of $E^{\mathrm{RC}}$ in terms of the two fixed isotherms in its construction. We provide an explanation of why the efficiency $\epsilon^{\mathrm{RC}}$ of $E^{\mathrm{RC}}$ is determined uniquely by the temperatures of its isotherms and nothing else, a hallmark of the Carnot theorem and a remarkable property of the reversible Carnot engine; see Conclusion 3 and Remark 7, which also explains why the efficiency is *independent of the working substance* of the engine (Protocol A). The entropy cancellation in Equation (5) is not the reason as it is generic to all cycles of any kind. Other reversible engines based on Protocol B so that they are different from $E^{\mathrm{RC}}$ are considered in detail in Section 7. In Section 7.1, we consider $E^{\mathrm{RNC}}$ and determine its efficiency. This engine paves the way towards considering a continuous distribution of heat mediums with temperatures ranging from $T_{0C}$ to $T_{0H}$. Their presence destroys the remarkable property of $E^{\mathrm{RC}}$ so the efficiency of this reversible engine is determined not by two fixed isotherms but by effective temperatures—see Claim 6—that are strongly *dependent on the working substance* because they follow the NonFix-protocol B. The efficiency $\epsilon^{\mathrm{RNC}}$ has the same form as $\epsilon^{\mathrm{RC}}$ in terms of the effective temperatures. We study $E^{\mathrm{ARC}}$ in Section 7.3, and show that its efficiency is also strongly dependent on the WS for the same reason as for $E^{\mathrm{RNC}}$, and can be put in the same form as $\epsilon^{\mathrm{RC}}$ in terms of the effective temperatures; see Claim 8.

All the above results are for reversible heat engines. We put forward Carnot’s argument for C-Th-2, point out its limitation, and extend it to all irreversible engines, not just the irreversible Carnot $E^{\mathrm{IrC}}$ engine in Section 8, and express the result in Theorem 1. We show that an engine must be specified not only by its protocol specifying the nature of its set of processes but also by its working substance in all cases; see Theorem 2. The most important result is the following:

**Conclusion** **1.** *The efficiency of any reversible engine E running under any protocol can be cast in a form that identifies a* fictitious *Carnot engine $E^{*\mathrm{RC}}$ in terms of effective hot and cold temperatures associated with its $P_{\mathrm{acc}}$ and $P_{\mathrm{rej}}$, respectively. The physics of effective temperatures is made clear in Section 7.2 so that there is* no *distinction in the physics of the two engines. Whether their efficiency is WS-dependent depends on their common protocol.*

The issue of regenerators is considered in Section 9 by focusing on the Stirling engine $E^{S}$, as there are major misconceptions and misunderstandings that one finds in the literature. Therefore, we try to obtain a handle on regenerators, where a non-Carnot (NC) efficiency $\epsilon_{\mathrm{NC}}>\epsilon$ in Equation (4) is often used instead of ϵ in Equation (2). We show that only for an ideal gas, $\epsilon_{\mathrm{NC}}^{\mathrm{RS}}=\epsilon^{\mathrm{RC}}$ in Equation (65) by treating a reversible regenerator, as is conducted by Kestin [16]. However, if this regenerator is replaced by a single or a fixed and finite number of heat exchangers, then they introduce irreversibility in the engine as proven in Theorem 3, which has no analog of a reversible engine. We also establish that the no-Carnot efficiency in Equation (73) fails to capture irreversibility so it should not be taken seriously as a measure of thermodynamic efficiency.

The last section provides a discussion and a brief summary of our new results.

## 2. Preliminaries

### 2.1. Temperature in Carnot’s Reflections [1]

The concept of temperature was well-established by the time Carnot wrote his *Reflections*, and was measured in the Fahrenheit (°F) and the Celsius (°C) scales. The absolute or the Kelvin scale was not yet invented, which is what we are going to be using in this study, as this provides a conjectural approach to derive the first law and the modern expression for the efficiency of the Carnot engine in Section 3. This also allows us to make contact with the modern use of the Kelvin scale temperature, denoted in this study by *T*, which is defined thermodynamically by ∂E/∂S in the standard notation. This helps to make our conceptual derivation mentioned above thermodynamically consistent, as we will see. It also allows us to treat the working system (WS) in an engine thermodynamically to obtain a handle on understanding the interplay between WS and the efficiency of the engine, one of our major goals mentioned earlier. Our conceptual approach is consistent with the use of the Carnot engine to obtain a WS-independent notion of the Kelvin temperature. As our interest is not to see how the absolute temperature scale came out of the work by Carnot, we refer the reader to a very interesting discussion of the history of temperature as part of an overview of classical thermodynamics by Saslow [19] in this journal.

Carnot uses the Celsius scale for the temperature. Most importantly, Carnot talks about the fall of caloric in terms of a temperature difference from a hot body to a cold body by using the waterfall analogy mentioned earlier; see Equation (1). Fortunately, this difference is the same in both the Celsius and the Kelvin scales, so we can use the difference in the absolute temperature instead of what we used in Equation (1), which plays an important role in Section 3.

With the choice of absolute scale temperature in this study, we absorb the Boltzmann constant in its definition to make *T* have the dimension of energy, just as the caloric *Q* and the motive power *W*. The entropy *S* now becomes a dimensionless number. This will be of tremendous use in carrying out dimensional analysis, as mentioned in the abstract.

### 2.2. Reversible Carnot Cycle (RC Cycle)

We have stated above the three different parts of, what is now known as the Carnot theorem, in his own words. The statement C-Th-1 is a precursor of Kelvin’s statement of the second law, and there is no need to modify it. In the modern literature, C-Th-2 and C-Th-3 are stated as follows; see, for example, the following [16]:

1. **C-Th-2M**: All heat engines operating between the same two heat mediums cannot have efficiencies greater than a reversible Carnot engine $E^{\mathrm{RC}}$ operating between the same mediums.
2. **C-Th-3M**: Every reversible heat engine operating between the same two heat mediums is equally efficient, regardless of WS employed or the operation details, and is equal to that of the Carnot engine $E^{\mathrm{RC}}$, which depends solely on the temperatures of its hot and cold heat mediums.

The phrase “between two heat mediums” that is commonly used in the literature to state the Carnot theorem has created much confusion. Here, we recall Fermi’s statement [20], a highly respected physicist, about a corollary of the Carnot theorem:

**Corollary** **1.** Fermi*’*s Corollary *: If there are several cyclic heat engines, some of which are reversible, operating around cycles between the same temperatures $T_{H}$ and $T_{L}$, all the reversible ones have the same efficiency, while the nonreversible ones have efficiencies which can never exceed the efficiency of the reversible engines.*

Seldman and Michalik [17] suggest that an authority such as Atkins [21], recognizing that a Stirling engine operates “between” the same two temperatures, must have the efficiency of a Carnot engine for all working substances but failed to provide any calculational support for it; see also Section 9.2. This clearly shows the importance of bringing in protocols, whose relevance for the Carnot theorem has not been fully appreciated.

It was the genius of Carnot to realize that to successfully convert heat to useful work (motive power), two macroscopically large but different heat mediums ${\tilde{\Sigma }}_{\mathrm{hH}}$ and ${\tilde{\Sigma }}_{\mathrm{hC}}$ at different temperatures $T_{0H}$ and $T_{0C}<T_{0H}$ are required, as shown in Figure 1, where an irreversible engine shown by Σ(t) at temperature T(t) accepts macroheat $\Delta_{e}Q_{H}$ from the hotter heat medium ${\tilde{\Sigma }}_{\mathrm{hH}}$, rejects macroheat $\Delta_{e}Q_{C}$ to the colder heat medium ${\tilde{\Sigma }}_{\mathrm{hC}}$, and performs exchange macrowork $W≐\Delta_{e}W$ on the work medium ${\tilde{\Sigma }}_{w}$ over the entire Carnot cycle. At his time, terms like isothermal and adiabatic were not part of thermodynamics so Carnot described his reversible cycle [1] (see p. 63 onwards) in seven steps involving four different reversible processes $P_{1},$ $P_{2},$$P_{3}$, and $P_{4}$, but the description has created a certain amount of confusion as to the nature of actual steps Carnot had in mind.

The macroheat accepted by the engine from the outside and denoted by $Q_{\mathrm{acc}}\equiv \Delta Q_{\mathrm{acc}}\equiv \Delta_{e}Q_{H}$ is to be treated as positive, and the macroheat rejected to the outside by $Q_{\mathrm{rej}}\equiv \Delta Q_{\mathrm{rej}}\equiv \Delta_{e}Q_{C}$ is to be treated as negative. But all temperatures are taken non-negative, as stated before.

**Remark** **1.** *The description of the seven steps is such that the system does not come back to the initial state after the end of $P_{4}$ during the first cycle [22]. In other words, the initial state *1* is to the right of $V_{1}$ with a slightly higher volume. This is because of the experimental setup that cannot determine the position $V_{4}$ so that the adiabat will heat up the system to have the exact value $V_{1}$. This problem does not occur in the protocol specification described in Section 2.4.*

**Figure 2:**
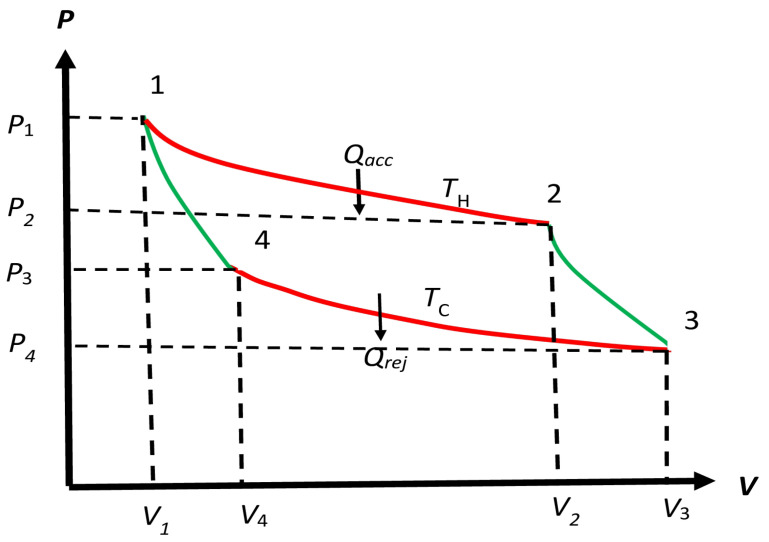
Schematic form of the cycle considered by Carnot, which starts from 1 and comes back to it as follows: 1→2→3→4→1. We will allow the four processes to be irreversible also for later use. The (reversible or irreversible) processes along 1→2 and 3→4 are isothermal at temperatures $T_{0H}$ and $T_{0C}<T_{0H}$, respectively. The (reversible or irreversible) processes along 2→3 and 4→1 are adiabatic (no macrohaet transfer) between temperatures $T_{0H}$ and $T_{0C}$, and $T_{0C}$ and $T_{0H}$, respectively. We will allow the temperature of the engine T(t) to be different from $T_{0H}$ and $T_{0C}$ along 1→2 and 3→4, respectively, for an irreversible engine. For a reversible engine, these processes remain isothermal as Carnot requires. The accepted macroheat $Q_{\mathrm{acc}}≐\Delta_{e}Q_{\mathrm{acc}}$ and rejected macroheat $Q_{\mathrm{rej}}≐\Delta_{e}Q_{\mathrm{rej}}$ occur along 1→2 and 3→4, respectively.

All steps were reversible, a term first used by Carnot to identify reversible processes. What is clear is that there are two distinct processes, $P_{1}:1\to 2$ and $P_{3}:3\to 4$, involving *isothermal* expansion and compression at $T_{0H}$ and $T_{0C}$, respectively, and two *adiabatic* processes, $P_{2}:2\to 3$ and $P_{4}:4\to 1$, involving expansion and compression to decrease and increase the temperature between the two isotherms, respectively. Whether the isothermal processes are isobaric or not is not clear from Carnot’s writing. Clapeyron [23] expanded on Carnot’s *Reflections* due to “reasonings difficult to apprehend, at results easily deducible from a more general law, …” He gave a rendition of the Carnot cycle for gases in the PV plane by considering isothermal processes as non-isobaric so the PV-macrowork can be deduced. The adiabatic processes along with this non-isobaric processes are shown in Figure 2, where $Q_{\mathrm{acc}}=\Delta_{e}Q_{H}$ is the exchange macroheat accepted from ${\tilde{\Sigma }}_{\mathrm{hH}}$, and $Q_{\mathrm{rej}}=\Delta_{e}Q_{C}$ is the exchange macroheat rejected to ${\tilde{\Sigma }}_{\mathrm{hC}}$. They represent *latent heats* along the isotherms. Claperyron takes them to have the same value in accordance with the caloric theory, but we will not require this equality as we will show that the equality is not needed for determining the efficiency of the Carnot cycle in Figure 2 in which mechanical macrowork is obtained “ … without the contact of bodies of different temperatures”, even though there is a caloric exchange between the two bodies (heat mediums), in the words of Clapeyron. Maxwell [24] also follows the same PV diagram. This diagram has been followed by most authors, so we will adopt it to be the *standard* diagram for any Carnot cycle, regardless of its working substance content. We will identify the Carnot engine by $E^{C}$, which stands for $E^{\mathrm{RC}}$ and $E^{\mathrm{IrC}}$, as the case may be; their cycle will be denoted as the Carnot cycle or simply as the C cycle.

### 2.3. Full Specification of Carnot Cycle

The complete specification of the Carnot engine described by its four different reversible processes $P_{1},$$P_{2},$ $P_{3}$, and $P_{4}$, as shown in Figure 2, requires only specifying its working substance (WS), two isotherm temperatures $T_{0H}$ and $T_{0C}$, volume $V_{1}$ of its initial macrostate $M_{1\mathrm{eq}}$ and the volume $V_{2}$ in macrostate $M_{2\mathrm{eq}}$, which arrived at the end of the hot isothermal expansion at $T_{0H}$.The left adiabat connects $V_{2}$ with $T_{0C}$ and uniquely determines $V_{3}$ and does not have to be specified. The cold isotherm then determines $V_{4}$ such that the left adiabat brings it back to the initial volume $V_{1}$. We also do not need to specify $V_{4}$. The pressures at each point of the cycle are determined uniquely by the equation of state of WS, and do not have to be specified separately. Thus, to completely specify the Carnot engine, we need to specify WS, $T_{0H}$ and $T_{0C}$, and $V_{1}$ and $V_{2}$. Thus, we must express $E^{\mathrm{RC}}$ as

(6a) $$E^{\mathrm{RC}}\left(\mathrm{WS},T_{0H},T_{0C},V_{1},V_{2}\right).$$

Instead of specifying $V_{2}$, we can also specify $Q_{\mathrm{acc}}$ during $P_{1}:1\to 2$, matching, which then determines $V_{2}$. In this case, we specify the engine as

(6b) $$E^{\mathrm{RC}}\left(\mathrm{WS},T_{0H},T_{0C},V_{1},Q_{\mathrm{acc}}\right).$$

Both specifications, also called protocols, are useful, as we will see. The first one is simple to use when talking about a single Carnot engine, while the second one is useful when considering many Carnot engines in parallel; see Section 5.

We now introduce the *Carnot ratios*, which do not appear in *Reflections* [1],

(7) $$\Delta R_{\mathrm{acc}}≐\frac{Q_{\mathrm{acc}}}{T_{0H}},\;\Delta R_{\mathrm{rej}}≐\frac{Q_{\mathrm{rej}}}{T_{0C}},$$

and which play a very important role in the analysis of the *interplay of WS and efficiency* of all heat engines, and not just the Carnot engine, as we now discuss in Section 2.4. Thus, instead of specifying $Q_{\mathrm{acc}}$ in Equation (6b), we can equally well specify $\Delta R_{\mathrm{acc}}$ in its place and express $E^{\mathrm{RC}}$ as $E^{\mathrm{RC}}($WS$,T_{0H},T_{0C},V_{1},\Delta R_{\mathrm{acc}})$.

### 2.4. Cycle Protocols and Working Substance

There are two different kinds of protocols to construct the cycle of a heat engine, which we will consider here.

A. **Fix-Protocol**: These protocols are *externally* forced from the heat and work mediums that then *determine the shape* of the cycle. As a result, WS responds to the protocol in this case by adjusting its macrostate according to the external mediums. As an example, the part of the protocol for the Carnot cycle specifying the two isotherms requires that WS adjusts its macrostates and determines the change $\Delta R_{P_{1}}$ during $P_{1}:1\to 2$ and $\Delta R_{P_{3}}$ during $P_{3}:3\to 4$. As we will see soon in Equation (20),
  (8a) $$\Delta R_{P_{1}}=-\Delta R_{P_{3}},$$
  a *remarkable* property of the cycle, which is the first clue to identifying equilibrium (EQ) entropy as a state function; see Section 3 and Section 4.2. Even though different WSs produce different $\Delta R_{P_{1}}$ and $\Delta R_{P_{3}}$, the above relation is always valid. Thus,
  (8b) $$Q_{\mathrm{acc}}=T_{0H}\Delta R_{P_{1}},Q_{\mathrm{rej}}=T_{0C}\Delta R_{P_{3}},$$
  which also vary from one WS to another. But it must be noted that for such a protocol, $T_{0H}$ and $T_{0C}$ remain the same for *all* working substances as they are externally fixed. This means that the ratio
  (8c) $${\left(Q_{\mathrm{rej}}/Q_{\mathrm{acc}}\right)}_{\mathrm{Fix}}\;\mathrm{is}\;\mathrm{independent}\;\mathrm{of}\;\mathrm{WS},$$
  where the suffix Fix refers to the above Protocol A. It is this particular property of the ratio that makes the efficiency of a Carnot engine independent of the WS macrostate $M_{1\mathrm{eq}}$ in the engine, and the four different reversible processes $P_{1},$$P_{2},$ $P_{3}$, and $P_{4}$, as shown in Figure 2. These processes include the two isotherms and the two adiabats, as explained in Section 2.2. The complete specification of its protocol is given in macrostate $M_{2\mathrm{eq}}$, arrived at after the isothermal expansion at $T_{0H}$.
B. **NonFix-Protocol**: These protocols *specify* the cycle, which then *determine the choice* of the necessary mediums such as the heat mediums $\left\{{\tilde{\Sigma }}_{\mathrm{hH}}\right\}$ and $\left\{{\tilde{\Sigma }}_{\mathrm{hC}}\right\}$. In this case, the temperatures of these heat mediums are determined by the WS of the engine that follows the specific cycle. Hence, the *distribution of temperatures* are dictated by WS. As we will see, the ratio $Q_{\mathrm{rej}}/Q_{\mathrm{acc}}$ now is controlled by the protocol
  (8d) $${\left(Q_{\mathrm{rej}}/Q_{\mathrm{acc}}\right)}_{\mathrm{NonFix}}\;\mathrm{is}\;\mathrm{dependent}\;\mathrm{on}\;\mathrm{WS}.$$

### 2.5. Arbitrary Cycle Characteristics

It is important to make more observations for any cycle, not just the one considered by Carnot. As the engine has come back to the starting point in both protocols A and B, the energy and entropy of the system (working substance) must return to its initial value, so their changes must be zero:

(9) $$\Delta E_{\mathrm{cyc}}\equiv 0,\Delta S_{\mathrm{cyc}}\equiv 0,$$

whether the cycle is reversible or irreversible.

It is useful to divide the entire cycle process $P_{\mathrm{cyc}}$ as made up of two distinct processes $P_{\mathrm{acc}}$ and $P_{\mathrm{rej}}$, during which the exchanged macroheat is accepted from and rejected to, respectively, the appropriate heat mediums; see Definition 1. They may include adiabats as part of them even though they do not allow any exchange macroheats. Using $\Delta_{e}Q_{H}$ and $\Delta_{e}Q_{C}$ over $P_{\mathrm{acc}}$ and $P_{\mathrm{rej}}$, respectively, we have

(10) $$\Delta_{e}Q_{\mathrm{cyc}}\equiv \Delta_{e}Q_{H}+\Delta_{e}Q_{C}<\Delta_{e}Q_{H},$$

assuming $\Delta_{e}Q_{C}\neq 0$. From this, we conclude that over $P_{\mathrm{acc}}$

(11) $$\Delta_{e}Q_{H}>\Delta_{e}Q_{\mathrm{cyc}}=\Delta_{e}W_{\mathrm{cyc}};$$

the last equality emerges if we use the first law, which Carnot was not privy to, and thus we will not use it when we follow Carnot. Following the first law, the efficiency as used by Carnot is

(12) $$\epsilon ≐\frac{\Delta_{e}W}{\Delta_{e}Q_{H}}<1$$

for any heat engine in both protocols A and B.

## 3. First Law and Entropy à la Carnot (Protocol A)

In this section, we wish to provide new arguments that show that based on what Carnot knew at his time and simple dimensional analysis, we can follow a logical thread, presumably not followed by Carnot himself, that leads to the cycle version of the first law and the first glimpse of the concept of EQ entropy, for which Clausius [2] usually gets credit as the first inventor. At the end of this section, it should become clear why we recommend calling entropy *Carnot–Clausius entropy*. Regarding the first law, we recall Carnot’s quote *Reflections*-1 and the discussion below it in Section 1.1 of the “presumptive evidence” for energy conservation by Carnot as offered by Kuhn [10] so that we should not be surprised by the discussion below of the first law and the evidence that we offer in its support. We must also recall [6] that Carnot, after abandoning the caloric theory, had estimated the mechanical equivalent of heat, so he regarded *W* and $Q_{\mathrm{acc}}$ to be both forms of energy. This makes ϵ *dimensionless*.

It follows from C-Th-3 that the efficiency of the Carnot engine (Protocol A), which is adimensional, can only depend on the adimensional ratio $\tau ≐T_{0C}/T_{0H}$ of the two isotherms

(13) $$\epsilon^{\mathrm{RC}}≐\frac{W}{Q_{\mathrm{acc}}}=\epsilon \left(T_{0C}/T_{0H}\right).$$

As Carnot was not yet familiar with the first law when he made the observation C-Th-3, he did not obtain the exact form of the function ϵ. Had he been aware of this law, he would have anticipated the concept of entropy also, as we will argue now by using the information available to Carnot at the time of writing *Reflections* [1], and by simply using dimensional analysis.But before we do that, we wish to argue that Carnot never had to use the two laws of thermodynamics to determine the engine efficiency.

### 3.1. Carnot’s Approach

While the caloric theory, according to which $\Delta_{e}Q_{\mathrm{cyc}}\equiv 0$, is mentioned at several places by Carnot [1], it is not involved in the definition of the efficiency of the reversible engine $E^{\mathrm{RC}}$ or its determination, as follows:

(14) $$\epsilon^{\mathrm{RC}}=\frac{\Delta_{e}W_{\mathrm{cyc}}}{\Delta_{e}Q_{H}}<\frac{\Delta_{e}W_{\mathrm{cyc}}}{\Delta_{e}Q_{\mathrm{cyc}}}\equiv 1,$$

where we have used Equation (10) for the first inequality, which is not mentioned by Carnot, but we have included it to show the problem with the caloric theory, for which the upper bound diverges to infinity. But this bound does not afflict Carnot’s computation as we describe below, where $Q_{\mathrm{cyc}}≐\Delta_{e}Q_{\mathrm{cyc}}\equiv 0$ is not used. The last identity is proved by using the conjectural Carnot approach, resulting in Equation (21).

As noted in Figure 2, $\Delta_{e}Q_{H}$ is simply denoted by $Q_{\mathrm{acc}}$, which is along the isothermal process 1→2 at fixed temperature $T_{0H}$, with P,V changing along it from $P_{1},V_{1}$ to $P_{2},V_{2}$. As there is no temperature change, we are dealing with latent heat. As Carnot was not aware of the first law, he could not use it. Thus, we also need to use an alternative method to determine the latent heat. For example, we could measure $Q_{\mathrm{acc}}$ by isothermal calorimetric technique that maintains a constant temperature throughout the isothermal process. Similarly, $\Delta_{e}W_{\mathrm{cyc}}$ is also amenable to measurement without ever invoking the first law.

**Claim** **2.** *Both exchange quantities in Equation (14) are easy to measure or determine for $E^{\mathrm{RC}}$ even without the use of the first law or the caloric theory.*

Therefore, Carnot’s conclusions are obtained without ever using the first law or invoking the caloric theory. This clearly shows the sheer ingenuity and deep understanding of the workings of heat engines that enabled Carnot to arrive at monumental results, given the incomplete information of the time. It must be clear that the determination of $\epsilon^{\mathrm{RC}}$ neither requires the first nor the second law, though its equivalence in terms of the temperatures of the two heat mediums, which Carnot does not obtain for good reasons, is easily obtainable by their use; we provide a simple derivation below.

### 3.2. First Law à la Carnot

The only two process macroheats for the entire heat cycle are the latent heats $Q_{\mathrm{acc}}$ and $Q_{\mathrm{rej}}$ along the two isotherms used in Equation (13). They determine another adimensional ratio

(15) $$\rho ≐Q_{\mathrm{rej}}/Q_{\mathrm{acc}}$$

over the cycle so it can also be used to determine the efficiency. The other possible ratio $W/(Q_{\mathrm{acc}}+Q_{\mathrm{rej}})$ is of quantities that are defined over the entire cycle so it cannot be identified as a ratio of quantities defined over individual isotherms. Therefore, the efficiency cannot be determined by it. Indeed, the latter ratio turns out to be unity and establishes the first law as we show below in Equation (21). Until we do so, we take

(16) $$W\propto Q_{\mathrm{acc}}+Q_{\mathrm{rej}},$$

with some unknown proportionality constant.

As ρ is also determined by the two isotherms, it must be an adimensional function of the above ratio τ:

(17) ρ≐ρ(τ).

All this can be concluded without the use of the first and the second laws.

The above discussion of Equations (15)–(17) is not what is found in *Reflections* [1] by Carnot, but he could have very easily concluded them even without knowing the two thermodynamic laws.

However, Carnot [1] (p. 61) did claim (see Erlichson [6] also) that

*Reflections*-3: “the motive power of heat depends also on the quantity of caloric used, and on what may be termed, on what in fact we will call, the *height of its fall*, that is to say, the difference of temperature of the bodies between which the exchange of caloric is made.”

We thus conclude, following Carnot and Erlichson, that

(18) $$W\propto Q_{\mathrm{acc}}\left(T_{0H}-T_{0C}\right).$$

Comparing it with Equation (16), Carnot could have concluded that

$$Q_{\mathrm{acc}}+Q_{\mathrm{rej}}\propto Q_{\mathrm{acc}}\left(T_{0H}-T_{0C}\right).$$

Taking the proportionality constant to be the inverse of $T_{0H}$ to yield $Q_{\mathrm{acc}}$ on both sides, we obtain

$$Q_{\mathrm{acc}}+Q_{\mathrm{rej}}=Q_{\mathrm{acc}}\left(1-T_{0C}/T_{0H}\right).$$

In other words,

(19) $$Q_{\mathrm{rej}}=-Q_{\mathrm{acc}}T_{0C}/T_{0H},$$

or

(20) $$Q_{\mathrm{acc}}/T_{0H}=-Q_{\mathrm{rej}}/T_{0C}$$

in terms of the Carnot ratios; see Equation (7). (Using the negative inverse of $T_{0C}$ also gives the same identity of the Carnot ratios.) It is quite possible that the relation between the two macroheats in Equation (19) may have given the motivation for Carnot to eventually abandon the caloric theory later on; see *Reflections*-1 again. But as we will see, he never had to use Equation (19) to prove C-Th in *Reflections*. We also observe from Equation (18) by using the proportionality constant $\beta_{0H}≐1/T_{0H}$ that

(21) $$W=Q_{\mathrm{acc}}\left(1-T_{0C}/T_{0H}\right)=Q_{\mathrm{acc}}+Q_{\mathrm{rej}}\equiv Q_{\mathrm{cyc}},$$

which is nothing but the *first law for a cycle*; see Equation (11), which is precisely the content of *Reflections*-1.

Incidentally, the equality in Equation (20) proves not only the equality in Equation (8a) but also satisfies the conclusion in Equation (8c) that the ratio ρ is independent of WS so all reversible Carnot engines $E^{\mathrm{RC}}$ have the same efficiency $\epsilon^{\mathrm{RC}}$, which we now derive.

As $\epsilon^{\mathrm{RC}}$ is determined by the entire cycle, it must be a function of the above ratio ρ. From Equation (19), we conclude that

(22) ρ=−τ,

and

(23) $$\epsilon^{\mathrm{RC}}=1-T_{0C}/T_{0H}.$$

The efficiency is independent of the choice of WS of $E_{\mathrm{RC}}$, and its four macrostates 1, 2, 3, and 4 forming a closed cycle $P_{\mathrm{cyc}}$, except for the ratio of the two isotherms; see the conclusion in Equation (8c) for Protocol A.

### 3.3. Entropy à la Carnot

We recall the definition of the Carnot ratios in Equation (7). The efficiency is independent of the choice of WS of $E^{\mathrm{RC}}$, and its four macrostates 1, 2, 3, and 4 forming a closed cycle $P_{\mathrm{cyc}}$, except for the ratio of the two isotherms. The form of the efficiency in Equation (23) simply implies that the ratio

(24) $$\Delta R≐Q/T_{0}$$

along the two isotherms 1→2 and 3→4 has the *same* magnitude but opposite signs along the two processes $P_{\mathrm{acc}}:1\to 2$ and $P_{\mathrm{rej}}:3\to 4$, respectively, regardless of the choice of 1, 2, 3, and 4 forming $P_{\mathrm{cyc}}$. As noted above, see Carnot’s quote *Reflections*-1 in Section 1.1; Carnot had eventually abandoned the caloric theory. As a consequence, $Q_{\mathrm{acc}}$ and $Q_{\mathrm{rej}}$ do not have the same magnitude, as would be the case in the caloric theory. This means that $\Delta R_{\mathrm{acc}}≐Q_{\mathrm{acc}}/T_{0H}$ denotes the difference R(2)−R(1) and $\Delta R_{\mathrm{rej}}$ denotes the difference R(4)−R(3) of a macroquantity *R* between the two end macrostates at 1 and 2, and 3 and 4, respectively. Along the two adiabats, where there are no macroheat exchanges, we can set $\Delta R_{\mathrm{adia}2}≐R\left(3\right)-R\left(2\right)=0$ along $P_{2}$ and $\Delta R_{\mathrm{adia}4}≐R\left(1\right)-R\left(4\right)=0$ along $P_{4}$. Using these values, we see that

(25a) $$\Delta R_{\mathrm{cyc}}≐\sum_{P_{k}}\Delta R_{k}≐\Delta R_{\mathrm{acc}}+\Delta R_{\mathrm{rej}}=0,$$

to establish that

(25b) $$\Delta R_{\mathrm{acc}}=-\Delta R_{\mathrm{rej}}$$

over the *entire cycle*. The discovery of this universal feature of ΔR by Carnot is the *precursor* of the eventual *recognition by Clausius* [2] that for any arbitrary thermodynamic *reversible* process along any closed path (cycle) $P_{\mathrm{rev},\mathrm{cyc}}$, as we show in Section 4.2, one can identify ΔR with EQ entropy change $\Delta S_{\mathrm{eq}}$, as follows:

(26) $$\Delta S_{\mathrm{eq}}\equiv \Delta R,$$

which follows from Equations (29a) and (29c).

**Remark** **2.** *Therefore, to be fair to Carnot’s anticipated contribution to the discovery of thermodynamic entropy S, we must refer to it as* Carnot–Clausius entropy*, although it is normally associated with Clausius alone. From now on, R and S will be treated as the same for this reason.*

**Remark** **3.** *As Equation (25a) satisfies the cycle condition on the entropy in Equation (9), Carnot engine comes back to the starting state; compare with Remark 1.*

**Remark** **4.** *It follows from Equation (25b) that there is a* complete cancellation *of the two entropy contributions in the ratio ρ to make it equal to (−τ), as shown in Equation (22).*

### 3.4. More on the Caloric Theory

We rewrite $\Delta_{e}Q_{H}$ and $\Delta_{e}Q_{C}$ in a form

(27) $$\frac{\Delta_{e}Q_{H}}{T_{H}}=-\frac{\Delta_{e}Q_{C}}{T_{C}}=\Delta R_{H}>0,$$

as $Q_{\mathrm{acc}}=\Delta_{e}Q_{H}>0$, and we are only dealing with non-negative temperatures. At this point, we turn to the caloric theory, according to which $\Delta_{e}Q_{H}=\left|\Delta_{e}Q_{C}\right|$. If valid, this will require $T_{0H}=T_{0C}$, which is mathematically invalid for the premise $T_{0H}>T_{0C}$. Therefore, the caloric theory needs to be rejected, which Carnot eventually conducted, as noted above.

What about reinterpreting Carnot’s writing as suggested by La Mer [12,13], which was reported earlier in Section 1.1. He suggested interpreting “calorique” as “entropy”. If we accept this reinterpretation, we will come to conclude that Carnot considered the ratio ρ in Equation (15) as the ratio of two entropy differences, which from Equation (25a) should be exactly equal to unity so the two macroheats in ρ must not depend on WSs. But macroheats are process quantities, actually *latent heats*, that depend directly on the temperature and entropy change, so they must be different along the two isotherms. Recall that Carnot had already abandoned the caloric theory, so he accepted the fact that ρ is not a simple constant equal to unity. This is also clear from Equation (22), according to which we must have, if we accept La Mer’s claim, $T_{0H}=T_{0C}$, which we have already rejected. As entropy is determined by the WS, this ratio must also depend on the WS, which would invalidate the Carnot conclusion in Conclusion 3. This would be a disservice to Carnot’s genius and would destroy his legacy if we accept La Mer’s reinterpretation. We do not find that the reinterpretation makes Carnot’s reasoning consistent.

**Remark** **5.** *It should be clear from the definition of ΔR in Equation (24) and its identification with ΔS in Equation (26) that the calorique of Carnot cannot be interpreted as entropy change. They obviously have different units: Q has the units of energy, and ΔS is dimensionless.*

## 4. Other Reversible Engines-I

### 4.1. Reversible Non-Carnot Cycle (RNC Cycle in Protocol B

**Figure 3:**
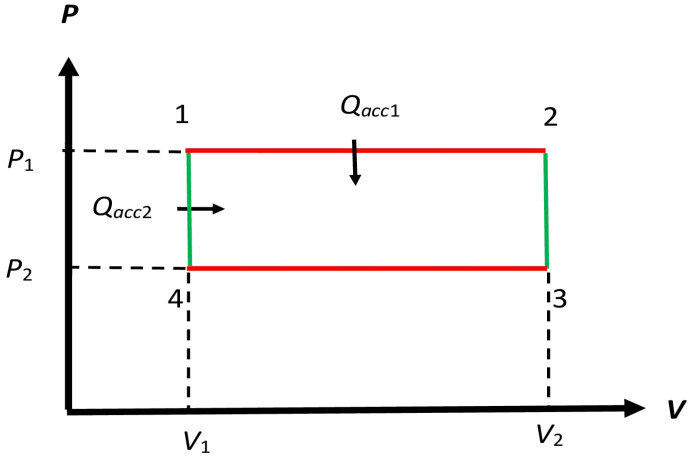
Schematic form of the cycle considered by Clapeyron, but with 2→3 and 4→1 as adiabatics shown in Figure 2. In that cycle, the accepted macroheat is only along 1→2 and 3→4, so $Q_{\mathrm{acc}1}≐\Delta_{e}Q_{\mathrm{acc}1}$ must be replaced by $Q_{\mathrm{acc}}$. We modify 2→3 and 4→1 to represent *isochoric* processes in our modified cycle for the reversible non-Carnot engine $E^{\mathrm{RNC}}$ in Section 4.1, so there is additional accepted macroheat $Q_{\mathrm{acc}2}≐\Delta_{e}Q_{\mathrm{acc}2}$ that occurs along 4→1, so the net accepted macroheat is $Q_{\mathrm{acc}}≐Q_{\mathrm{acc}1}+Q_{\mathrm{acc}2}$ in the modified engine. We will only consider reversible modified engine $E^{\mathrm{RNC}}$.

Clapeyron [23] (see Figure 2) also considers a liquid with its vapor in equilibrium as the WS for the Carnot engine and proposes a PV diagram with isobaric-isothermal processes along $P_{1}$ and $P_{3}$, similar to that shown in Figure 3, but still allows for adiabatic expansion and contraction along $P_{2}$ and $P_{4}$, respectively, as in Figure 2, to keep up with the spirit of a Carnot cycle; see a delightful discussion on these issues by Tanajö [22]. In any case, this cycle representation does not follow the protocol requirement laid out by Carnot; see Carnot’s quote *Reflections*-2 in Section 1.2. Thus, we identify such a cycle as a non-Carnot (NC) cycle. However, we will replace the latter processes $P_{2}$ and $P_{4}$ by isochoric processes, as shown in Figure 3, for a non-Carnot (NC) engine $E^{\mathrm{RNC}}$ to make a very important point—see Section 7.1—which has not been made, to the best of our knowledge. Such isochoric processes are parts of the Otto and Stirling cycles.

The processes in Figure 3 for $E^{\mathrm{RNC}}$ require the temperature to change isochorically from $T_{0H}$ to $T_{0C}$ and vice versa. To make these processes reversible requires a set of additional heat mediums $\left\{\Sigma_{kh}\right\}$ with fixed temperatures $\left\{T_{k0}\right\}$ that range from $T_{0H}$ to $T_{0C}$ [16] (Section 5.14.1). The protocol here belongs to Protocol B in Section 2.4 because the choice of $\left\{T_{k0}\right\}$ is dictated by the WS of the engine. By bringing the engine in contact with these heat mediums, we can let the engine reversibly make the transitions 2→3 and 4→1. During these transitions, macroheat $Q_{\mathrm{acc}2}$ is accepted from 4→1 by the engine so the net accepted macroheat is the sum of $Q_{\mathrm{acc}1}$ and $Q_{\mathrm{acc}2}$ as shown in Figure 3. A similar macroheat rejection occurs along 2→3→4, which is not shown. This suggests diving the entire cycle process $P_{\mathrm{cyc}}$ as made up of two distinct processes $P_{\mathrm{acc}}$ and $P_{\mathrm{rej}}$ during which exchange macroheat is accepted and rejected, respectively, to the appropriate heat mediums.

### 4.2. Arbitrary Reversible Cycle (ARC Cycle) in Protocol B and Clausius Approach

**Figure 4:**
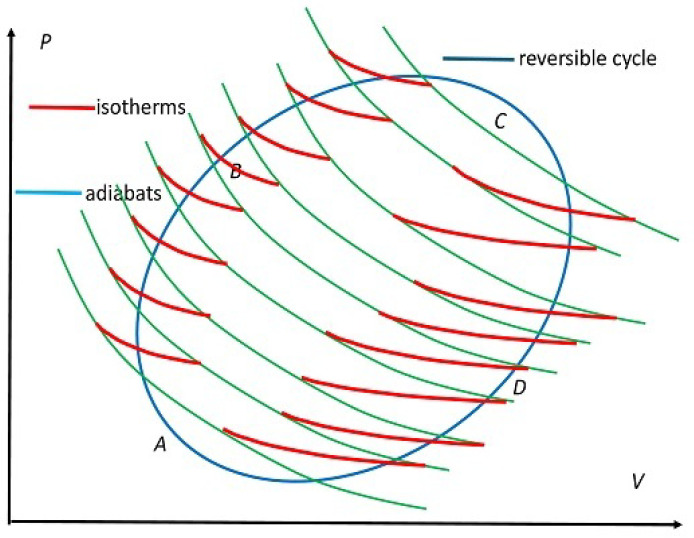
Schematic form of replacing an arbitrary reversible cycle shown in blue by a series of small but exaggerated-sized RC cycles placed next to each other. The red segments represent isotherms at infinitesimally different temperatures and green curves denote adiabats sharing consecutive C cycles. The segment ABC represents $P_{\mathrm{acc}}$, along which $\Delta_{e}Q_{\mathrm{acc}}$ is accepted and the segment CDA represents $P_{\mathrm{rej}}$, along which $\Delta_{e}Q_{\mathrm{rej}}$ is rejected.

We now justify the identification in Equation (26) of *R* and *S* and Remark 2. Thanks to Clausius [2], we know how any *arbitrary reversible* cycle (ARC cycle) $P_{\mathrm{ARC}}$ shown in blue in Figure 4 can be turned into a large number of RC cycles, with their isothermal temperatures shown by red segments differing from those of their neighboring cycles by *infinitesimal* amounts. This requires as above a sequence $\left\{\Sigma_{j\mathrm{hH}},\Sigma_{j\mathrm{hC}}\right\}$ of heat mediums with *fixed* temperatures $\left\{T_{j0H},T_{j0C}\right\}$ to represent the isothermal red segments of temperatures $\left\{T_{jH},T_{jC}\right\}$ of $\left\{j\right\}$th RC cycles along $\left\{P_{\mathrm{acc}},P_{\mathrm{rej}}\right\}$ of $P_{\mathrm{ARC}}$, as shown in Figure 4. We see again that the choice $\left\{T_{j0H},T_{j0C}\right\}$ is dictated by the WS of the cycle, so we are considering Protocol B here. Because of the decomposition into RC cycles, we have added C into ARC as a reminder to denote this particular cycle; see also Kestin [16] (Section 10.6.4).

For reversibility, we require the equality

(28a) $$\left\{T_{jH},T_{jC}\right\}=\left\{T_{j0H},T_{j0C}\right\},\forall j.$$

We use *t* as a continuous analog of *j* to denote time as the cycle $P_{\mathrm{ARC}}$ is completed. The continuum analog of the above equation becomes

(28b) $$T_{jH}\left(t\right)=T_{0H}\left(t\right)\;\mathrm{over}\;\mathrm{ABC},T_{jC}\left(t\right)=T_{0C}\left(t\right)\;\mathrm{over}\;\mathrm{CDA}.$$

The adiabats in green are shared by neighboring RC cycles so that they are traversed in opposite directions with their contributions to the ARC cycle cancelling out. This decomposition allowed Clausius to use the Carnot ratio to identify EQ entropy change

(29a) $$dS_{\mathrm{eq}}≐d_{e}Q\left(t\right)/T_{0}\left(t\right)=dR$$

following Equation (7) and to demonstrate the EQ entropy function (note that in EQ, $dQ\left(t\right)=d_{e}Q\left(t\right),d_{i}Q\left(t\right)=0$ so $dR=d_{e}Q\left(t\right)/T_{0}\left(t\right)$) as a *state function*

(29b) $$\Delta S_{\mathrm{cyc}}=\oint_{P_{\mathrm{ARC}}}d_{e}Q\left(t\right)/T_{0}\left(t\right)=\oint_{P_{\mathrm{ARC}}}dQ\left(t\right)/T\left(t\right)=0$$

in terms of the continuum picture in Equation (28b). The derivation refers to the entropy of an arbitrary reversible cycle so the entropy change above refers to the EQ change

(29c) $$dS_{\mathrm{eq}}=d_{e}Q\left(t\right)/T_{0}\left(t\right)=dQ\left(t\right)/T\left(t\right)\equiv dR,$$

which finally justifies the identification in Equation (26). It is clear from the construction how $P_{\mathrm{acc}}$ and $P_{\mathrm{rej}}$ with changing (*non-isothermal*) temperatures T(t) as the WS keeps adjusting its macrostates; see Equation (8d) for Protocol B, as we move along ABC and CDA in Figure 4, respectively, are replaced by a sequence of step-wise fixed (*isothermal*) red temperature segments belonging to $\left\{j\right\}$th RC cycles. For the ARC cycle, T(t) in the continuum limit must match exactly with changing temperatures of ${\tilde{\Sigma }}_{\mathrm{hH}}$ and ${\tilde{\Sigma }}_{\mathrm{hC}}$ over $P_{\mathrm{acc}}$ and $P_{\mathrm{acc}}$ to ensure *reversible* macroheat exchanges $\Delta_{e}Q_{H}$ and $\Delta_{e}Q_{C}$, respectively, over the entire cycle.

To appreciate the significance of Clausius construction for our investigation, we make the following observation. Let $E_{j}^{\mathrm{RC}}$ denote one of the possible infinitesimal RC cycles in the construction, with *j* now indexing them from the minimum efficiency $\epsilon_{\min}^{\mathrm{RC}}$ to maximum efficiency $\epsilon_{\max}^{\mathrm{RC}}$ as j=1,2,⋯ increases. Then, we have

$$\Delta_{e}W_{\mathrm{cyc}}^{\mathrm{ARC}}=\sum_{j}d_{e}W_{j\mathrm{cyc}}^{\mathrm{RC}},\Delta_{e}Q_{\mathrm{acc}}^{\mathrm{ARC}}=\sum_{j}d_{e}Q_{j\mathrm{acc}}^{\mathrm{RC}},$$

with

(30) $$\epsilon_{j}^{\mathrm{RC}}=\frac{d_{e}W_{j\mathrm{cyc}}^{\mathrm{RC}}}{d_{e}Q_{j\mathrm{acc}}^{\mathrm{RC}}}$$

for $E_{j}^{\mathrm{RC}}$; note that $\Delta_{e}Q_{\mathrm{acc}}^{\mathrm{ARC}}\to \Delta_{e}Q_{H}=Q_{\mathrm{acc}}$ and $\Delta_{e}W_{\mathrm{cyc}}^{\mathrm{ARC}}\to \Delta_{e}W=W$ in the continuum limit j→∞.

**Remark** **6.** *It is clear that the efficiency*

$$\epsilon^{\mathrm{ARC}}=\frac{\Delta_{e}W_{\mathrm{cyc}}^{\mathrm{ARC}}}{\Delta_{e}Q_{\mathrm{acc}}^{\mathrm{ARC}}}$$

*of the entire ARC cycle—see Equation (2)—obeys the inequality*

(31) $$\epsilon_{\min}^{\mathrm{RC}}\leq \epsilon^{\mathrm{ARC}}\leq \epsilon_{\max}^{\mathrm{RC}}.$$

**Claim** **3.** *As ARC cycle $P_{\mathrm{ARC}}$ is the most generic reversible cycle in the PV plane, we claim that any reversible cycle, some of which have been introduced already, can be replaced by a collection of RC cycles. Therefore, many but not all of the results for RC cycles also hold for an ARC cycle, as discussed later.*

The importance of the Clausius construction that closely follows the clue embedded in the Carnot ratio in Equation (7) in the development of classical thermodynamics is without reproach. This alone justifies extending RC cycles to also cover the situation in which $T_{0H}\left(V\right)$ and $T_{0C}\left(V\right)$ change over $P_{1}$ and $P_{3}$, respectively, as a function of *V*, so they become *non-isothermal*, following Protocol B. In contrast, they are isothermal in RC cycles in which the temperatures remain *fixed* by the external mediums; see Equation (8c) for Protocol A. The other two segments, $P_{2}$ and $P_{4}$, remain adiabatic in both cycles. Therefore, we will also consider this extension of the RC cycle, which we denote in short by the RC–B cycle (nF: not fixed as a reminder of Protocol B), and the corresponding engine we denote by $E^{\mathrm{RCnF}}$ to make a distinction with $E^{\mathrm{RC}}$. The Clausius construction provides a means to handle the case of heat mediums with nonfixed temperatures $T_{0H}\left(V\right)$ and $T_{0H}\left(V\right)$ that modifies the original construct (Protocol A) of the RC cycle by Carnot, but still leaves it as a reversible cycle. We will use this modified engine $E^{\mathrm{RCnF}}$ later to make an important point about the interplay of WS and protocols; see Remark 8 and Equation (8d).

## 5. Two Carnot Cycles in Parallel in Protocol A

### 5.1. Protocol Setup

Before making the important point mentioned above for the case of having a continuum of heat mediums, we wish to demonstrate that the situation is very different when we consider a finite sequence of reversible Carnot engines, as we describe now. Consider two reversible Carnot engines $E_{1}^{\mathrm{RC}}$ and $E_{2}^{\mathrm{RC}}$ containing the same WS in parallel to form the combined engine $E^{\mathrm{RC}2}$, which we show follows Protocol A; as an example, consider two neighboring reversible Carnot engines such as $E_{j}^{\mathrm{RC}}$, with *j* and j+1 in Figure 4. The engines share an inner adiabatic portion common to both, which is traversed in opposite directions, so this portion contributes nothing to the combined engine $E^{\mathrm{RC}2}$, provided both engines use the same WS; we assume this to be the case. Then this portion can be removed. This leaves behind two small inner adiabatic segments, one connecting the two hot temperature isotherms at $T_{01H}$ and $T_{02H}$ and the other one connecting the two cold temperature isotherms at $T_{01C}$ and $T_{02C}$. The two outer adiabats connect $T_{01H}$ with $T_{01C}$ and $T_{02H}$ with $T_{02C}$, respectively.

The cycle for each engine requires specifying its working substance, two isotherms, its initial volume $V_{1}$, and the volume $V_{2}$ at the end of the hot isotherm at $T_{0H}$; see Section 2.3. However, completely specifying $E^{\mathrm{RC}2}$ does not require twice the information, as we now discuss. We use the protocol in Equation (6b) for $E_{1}^{\mathrm{RC}}$, which determines the intermediate volume $V_{1}^{*}$ at the end of the first hot isotherm $T_{01H}$ by the value $Q_{1\mathrm{acc}}$ specified for $E_{1}^{\mathrm{RC}}$. The intermediate adiabat portion then connects $T_{01H}$ with $T_{02H}$ and determines the second intermediate volume $V_{2}^{*}$ that is the starting volume of $E_{2}^{\mathrm{RC}}$ on its hot isotherm $T_{02H}$. We now *demand* as part of the protocol for $E^{\mathrm{RC}2}$ that

$$\Delta R_{2H}=\alpha \Delta R_{1H}$$

where α is a fixed constant, which we will take to be unity for simplicity, and where $\Delta R_{2H}=Q_{2\mathrm{acc}}/T_{02H}$ and $\Delta R_{1H}=Q_{1\mathrm{acc}}/T_{01H}$. Thus, we demand

$$\Delta R_{2H}=\Delta R_{1H}.$$

This now determines $V_{2}$, the volume at the end of the last hot isotherm of $E^{\mathrm{RC}2}$. At this point, we have

(32) $$\Delta R_{H}=\Delta R_{1H}+\Delta R_{2H}.$$

We now use the right adiabat to connect $T_{02H}$ to $T_{02C}$ to determine the right end volume of $E^{\mathrm{RC}2}$ at the cold isotherm $T_{02C}$. This does not change the value of $\Delta R_{H}$. We now follow the cold isotherm for compression by rejecting $Q_{2\mathrm{rej}}≐$$T_{02C}\Delta R_{2C}$, with the requirement that

$$\Delta R_{2C}=-\Delta R_{2H}$$

so that $Q_{2\mathrm{rej}}$ is a known quantity. This determines the intermediate volume $V_{3}^{*}$ at the left end of the cold isotherm $T_{02C}$. We now connect this isotherm to $T_{01C}$ by the intermediate adiabat without changing $\Delta R_{2C}$, and obtain the next intermediate volume $V_{4}^{*}$ at the right end of the cold isotherm $T_{01C}$. At this point, we isothermally compress $T_{01C}$ to reject $Q_{1\mathrm{rej}}≐T_{01C}\Delta R_{1C}$, determined by

$$\Delta R_{1C}=\Delta R_{2C},$$

and arrive at volume $V_{4}$, which is at the left of this isotherm. Observe that the conditions imposed on all macroheats other than the initial macroheat $Q_{1\mathrm{acc}}$ has determined the complete protocol specification (WS, $V_{1},Q_{1\mathrm{acc}}$, four isotherms) of the cycle for the combined engine $E^{\mathrm{RC}2}$. All that is left now is to bring the engine back to the initial EQ macrostate at $V_{1}$ by connecting the last macrostate at $V_{4}$ by the left-most adiabat. Note that the sum of ΔR over the entire cycle vanishes as expected of the entropy

(33) $$\Delta R_{1H}+\Delta R_{2H}+\Delta R_{2C}+\Delta R_{1C}=0,$$

which is consistent with Equation (25a). As the cycle condition is satisfied, our protocol with the four fixed isotherms (Protocol A) has brought the engine back to the starting point; see Remark 3. Therefore, our protocol has avoided the incomplete cycle of Carnot; see Remark 1.

### 5.2. Efficiency and Effective Temperature

The efficiency of $E^{\mathrm{RC}2}$ is now easily found

(34a) $$\epsilon^{\mathrm{RC}2}=1+\left(Q_{1\mathrm{rej}}+Q_{2\mathrm{rej}}\right)/\left(Q_{1\mathrm{acc}}+Q_{2\mathrm{acc}}\right)$$

(34b) $$=1-\left(T_{01C}+T_{02C}\right)/\left(T_{01H}+T_{02H}\right),$$

which is *uniquely* determined by the protocol of $E^{\mathrm{RC}2}$ in terms of the temperatures of the external mediums, so its value is not affected by changing to any other WS; see Equation (8c). This is possible as the net entropy change $\Delta R_{H}≐\Delta R_{1H}+\Delta R_{2H}$ over the hot isotherms cancels out with $\Delta R_{C}≐\Delta R_{1C}+\Delta R_{2C}$ in accordance with the entropy conservation in Equation (33), just as it happens for a single $E^{\mathrm{RC}}$ as we see in Equation (25b) and the discussion following it; see Remark 4. However, this cancellation is not what ensures the independence of $\epsilon^{\mathrm{RC}2}$ on WS; it is due to the isotherms being controlled by external heat mediums that are unrelated to WSs.

If we had taken an arbitrary α, the efficiency would also depend on it, as can be easily seen. The argument can be easily extended to any finite number ν<∞ of Carnot cycles forming engines $E^{\mathrm{RC}\nu }$ in parallel with the same conclusion, as follows:

**Conclusion** **2.** *The efficiency of any reversible engine $E^{\mathrm{RC}\nu }$ composed of a* finite *number ν<∞ of reversible Carnot cycles is independent of WS, as the involved isotherms are controlled by mediums with fixed temperatures (Protocol A) that directly determine the shape of its cycle. During the entire heat cycle, all WSs keep adjusting their macrostates to match the same fixed isotherms with the result of having no effect on the efficiency of the resulting heat engine. The cancellation of the two entropy terms is not responsible for this independence, as* the cancellation is a generic property of all cycles*, as we will soon see.*

It should be obvious that we can identify a hypothetical Carnot engine $E^{*\mathrm{RC}}$ of efficiency

(35) $$\epsilon^{*\mathrm{RC}\nu }≐\epsilon^{*\mathrm{RC}}=1-T_{0C}^{*}/T_{0H}^{*},$$

where we have introduced the effective temperatures

(36) $$T_{0C}^{*}≐\frac{1}{\nu }\sum_{l=1}^{\nu }T_{0lC},T_{0H}^{*}≐\frac{1}{\nu }\sum_{l=1}^{\nu }T_{0lH}.$$

The physics of effective temperatures are postponed to Section 7.2.

## 6. Carnot’s Engine Efficiency $\epsilon^{\mathrm{RC}}$: Modern Approach

We have already determined the efficiency of $E^{\mathrm{RC}}$ in Equation (23) by using the arguments that Carnot could have used. We now show that the derivation there was *completely logical* and *correct* by using the modern approach post-Carnot. On a TS plane, the reversible Carnot cycle [1] in Figure 2 appears as a rectangle, with $P_{1}$ and $P_{2}$ replaced by $T_{0H}$ and $T_{0C}$, respectively, and $V_{1}$ and $V_{2}$ replaced by $S_{1}$ and $S_{2}$, respectively. The reversible non-Carnot cycle (RNC cycle) in Figure 3, however, does not look similar to the RC cycle, except that it also has four segments. The corresponding engine is denoted by $E^{\mathrm{RNC}}$. It is clear that one can design any number of reversible cycles by specifying what different processes $P_{1},$$P_{2},$$P_{3}$, and $P_{4}$ represent depending on the protocols A and B introduced in Section 2.4. As required for any reversible cycle, all its segments are reversible, so there is no irreversibility

(37) $$\Delta_{i}S_{\mathrm{cyc}}\equiv 0,\Delta_{i}W_{\mathrm{cyc}}\equiv 0,\Delta_{i}Q_{\mathrm{cyc}}\equiv 0.$$

We will later consider all of these collectively, when we derive Equation (49).

The reversible Carnot cycle in Figure 2 appears as a closed loop in the PV plane and is executed in a clockwise manner; see [1] for details, but also see [5]. The exchange or the useful macrowork $\Delta_{e}W_{\mathrm{cyc}}$ is the area of the cycle in the PV plane, performed by the engine during the entire reversible cycle on the outside work medium ${\tilde{\Sigma }}_{w}$.

As noted in Figure 3, $\Delta_{e}Q_{H}$ now has two parts. The first part $Q_{\mathrm{acc}1}$ represents the latent heat, as discussed above, along the isothermal process 1→2 at fixed temperature and pressure $T_{0H}$ and $P_{1}$, respectively, and can be measured. The other part $Q_{\mathrm{acc}4}$ is along the isochoric process 4→1 at fixed volume $V_{1}$, as the pressure changes from $P_{2}$ to $P_{1}$ and the temperature changes from $T_{0C}$ to $T_{0H}$. It is here that we need the continuous sequence of heat mediums with temperatures between $T_{0H}$ and $T_{0C}$, as discussed in Section 4.2 to ensure that macroheat exchanges remain reversible along the isochore. We now use the state variable T(P,V) obtained from the equation of the state of WS used in the engine as the integration variable along the left isochore, and use the heat capacity $C_{V}\left(T,V_{1}\right)$ at constant volume to determine $Q_{\mathrm{acc}4}$ by integrating

(38) $$Q_{\mathrm{acc}4}=\int_{4\to 1}C_{V}\left(T,V_{1}\right)dT.$$

As $Q_{\mathrm{acc}4}$ requires the heat capacity of WS, a different working substance would result in different values of $Q_{\mathrm{acc}}$, while the exchange or useful macrowork $\left(P_{1}-P_{2}\right)\left(V_{2}-V_{1}\right)$, is determined by parameters of the cycle controlled from the outside so it is independent of the properties of WS. This is because it follows Protocol B. Thus:

**Claim** **4.** *Even though we have a reversible NC engine, its efficiency is* not *working substance independent because of Equation (8d). We will see below that the efficiency of an RC engine is truly independent of WS used in the engine because of Equation (8c).*

### 6.1. Using the First Law

After any cycle (reversible or not, Protocol A and B) is completed, we have from Equation (9)

(39) $$\Delta E_{\mathrm{cyc}}=\Delta_{e}W_{\mathrm{cyc}}-\Delta_{e}Q_{\mathrm{cyc}}=0,\Delta S_{\mathrm{cyc}}=0;$$

here, $\Delta_{e}Q_{\mathrm{cyc}}\equiv$$\Delta_{e}Q_{H}+\Delta_{e}Q_{C}$, and $\Delta S_{\mathrm{cyc}}$ is the entropy change over the complete cycle; see Equation (9). We recognize that $\Delta E_{\mathrm{cyc}}=0$ above is nothing but a justification of Equation (21), which Carnot could have concluded after the abandonment of the caloric theory. We have extended the definition of Carnot efficiency in Equation (14) to any engine in Equation (12), which we now write as

(40) $$\epsilon ≐1+\frac{\Delta_{e}Q_{C}}{\Delta_{e}Q_{H}}<1,$$

where we have used $\Delta_{e}W_{\mathrm{cyc}}=\Delta_{e}Q_{\mathrm{cyc}}$ from the first law. The inequality justifies the inequality in Equation (14) obtained in the Carnot approach without using the first law and merely results from the observation that $\Delta_{e}Q_{C}<0$ and $\Delta_{e}Q_{H}>0$.

**Claim** **5.** *It should be recognized that $\Delta_{e}Q_{H}$ and $\Delta_{e}Q_{C}$ are process quantities so their values depend strongly on the WS of the engine, hence their ratio in Equation (15)*

(41) $$\rho ≐\frac{\Delta_{e}Q_{C}}{\Delta_{e}Q_{H}},$$

*in their terms also depends strongly on the WS of the engine. Therefore, the efficiency ϵ of the engine also depends strongly on the WS; however, compare with Conclusion 2.*

### 6.2. RC Engine $E^{\mathrm{RC}}$

The conventional textbook version of the Carnot engine $E^{\mathrm{RC}}$ discussion is as follows. The macrowork $\Delta_{e}W_{\mathrm{cyc}}$ is the area under the curve of the cycle in the PV plane. There is no macroheat exchange along $P_{2}$ and $P_{4}$ so they contribute nothing to ΔS. The accepted latent macroheat $\Delta_{e}Q_{H}=T_{0H}\left(S_{2}-S_{1}\right)$ is along $P_{1}$, and the rejected latent macroheat $\Delta_{e}Q_{C}=T_{0C}\left(S_{4}-S_{3}\right)$ is along $P_{3}$. From the last equation $\Delta S_{\mathrm{cyc}}\equiv 0$ in Equation (39), we find that

(42) $$S_{2}-S_{1}=S_{3}-S_{4},$$

which is nothing but Equation (25b). We now use Equation (40) to finally obtain the standard result

(43) $$\epsilon^{\mathrm{RC}}=1-\frac{T_{0C}}{T_{0H}},$$

a result already obtained earlier with the Carnot approach in Equation (23). This efficiency is generalized in Equation (49) to $E^{\mathrm{RNC}}$. However, the efficiency of $E^{\mathrm{RC}}$ has a remarkable form as determined only by parameters $T_{0H}$ and $T_{0C}$ of the cycle that are controlled from outside the system, which we paraphrase as

**Conclusion** **3.** *Because of the* exact cancellation *of entropy differences, which are determined by the WS of the engine, in the ratio ρ, the efficiency of $E^{\mathrm{RC}}$ is* independent *of WS only because $T_{0H}$ and $T_{0C}$ are determined by the external mediums oblivious of any WS. This should be contrasted with Claim 5.*

This conclusion was also obtained partly in Section 3 as Remark 4 and formalized as Conclusion 2 by our conjectural reasoning, which the modern understanding validates as completely logical. However, the entropy cancellation is not the source of WS independence, as the former is deeply rooted in the cycle property $\Delta S_{\mathrm{cyc}}\equiv 0$ of entropy—see Equation (5)—which is a *generic property* of $P_{\mathrm{acc}}$ and $P_{\mathrm{rej}}$ of all engines, even if their efficiencies depend on their WSs.

**Remark** **7.** *Carnot was most certainly not familiar with the concept of entropy. Therefore, it is not clear how Carnot arrived at the extraordinary conclusion that $\epsilon^{\mathrm{RC}}$ is independent of WS. We must attribute this to his deep understanding of thermodynamics and his genius that enabled him to make this* fundamental *observation about his reversible engine $E^{\mathrm{RC}}$, which we call the* Carnot Observation.

The brilliance of this observation is further strengthened by the fact that this is not true of any other *reversible engines*, such as $E^{\mathrm{RNC}\;}$, which are not a reversible Carnot engine $E^{\mathrm{RC}}$, as we will demonstrate in Section 7.

## 7. Other Reversible Engines with Effective Temperatures

### 7.1. RNC Engine $E^{\mathrm{RNC}}$ (Protocol B)

As this reversible engine in Figure 3 has no adiabatic processes that are an integral aspect of the Carnot engine $E^{\mathrm{RC}}$, as discussed in the previous section, we have identified it as a non-Carnot engine. We first focus on its segments 1→2 and 4→1, over which $\Delta_{e}Q_{H}≐Q_{\mathrm{acc}1}+Q_{\mathrm{acc}4}$ is accepted by $E^{\mathrm{RNC}}$. Recall that we need the continuous sequence of heat mediums [16] with temperatures between $T_{0H}$ and $T_{0C}$, as discussed in Section 4.2 to ensure that macroheat exchanges remain reversible along the isochore along 4→1. We use the state variable S(T,V) as an integration variable below. Along the isothermal segment 1→2, we obtain

(44a) $$Q_{\mathrm{acc}1}=\int_{1\to 2}TdS=T_{0H}\left(S_{2}-S_{1}\right),$$

and along the isochore (but non-isothermal) segment 4→1, we have

(44b) $$Q_{\mathrm{acc}4}=\int_{4\to 1}TdS\equiv T^{*}\left(S_{1}-S_{4}\right),$$

where we have used the mean-value theorem of calculus to introduce an intermediate temperature $T^{*}$ that lies between $T_{0H}$ and $T_{0C}$. We thus find that along the process $P_{\mathrm{acc}}$ identified by 4→1→2, we can express $\Delta_{e}Q_{H}$ as

(45) $$\Delta_{e}Q_{H}=\int_{P_{\mathrm{acc}}}TdS=T_{0H}^{*}\left(S_{2}-S_{4}\right)>0,$$

in terms of another intermediate temperature $T_{0C}<T_{H}^{*}<T_{0H}$ between $T_{0C}$ and $T_{0H}$ over $P_{\mathrm{acc}}$. From the definitions of

(46) $$T^{*}≐\frac{Q_{\mathrm{acc}4}}{S_{1}-S_{4}},T_{0H}^{*}≐\frac{\Delta_{e}Q_{H}}{S_{2}-S_{4}}$$

given above, it is clear that the two intermediate temperatures are not necessarily the same. We also observe that for positive $T_{0H}^{*}$,

(47) $$S_{2}>S_{4}$$

to ensure positive $\Delta_{e}Q_{H}$; we do not consider negative temperatures in this study. We similarly find that over the process $P_{\mathrm{rej}}$ identified by 2→3→4, we can express $\Delta_{e}Q_{C}$ as

(48) $$\Delta_{e}Q_{C}=\int_{P_{\mathrm{rej}}}TdS=T_{0C}^{*}\left(S_{4}-S_{2}\right)<0,$$

in terms of another intermediate temperature $T_{0C}<T_{0C}^{*}<T_{0H}$ between $T_{0C}$ and $T_{0H}$ over over $P_{\mathrm{rej}}$. We again observe that (positive $T_{0C}^{*}$) the inequality in Equation (47) remains satisfied.

**Claim** **6.** *Effective temperatures are determined by process quantities under integrals so they strongly depend on the WS unless we deal with isothermal processes; see Equation (8d). This should be contrasted with the Clausius observation in Conclusion 3, Remark 7, and Equation (8c).*

Using the general definition of efficiency in Equation (40), we now obtain $\epsilon^{\mathrm{RNC}}$ for the RNC engine

(49) $$\epsilon^{\mathrm{RNC}}=1-\frac{T_{0C}^{*}}{T_{0H}^{*}}<1-\frac{T_{0C}}{T_{0H}}<1;$$

From the inequality, it immediately follows that inequalities

(50) $$T_{0C}<T_{0C}^{*}<T_{0H}^{*}<T_{0H}$$

are always satisfied. This thus proves the following

**Claim** **7.** *The standard formulation of the efficiency of $E^{\mathrm{RNC}}$ that follows Protocol B in terms of its hot and cold “effective” temperatures appears similar to the efficiency of $E^{\mathrm{RC}}$ in Equation (43), except that the efficiency is not independent of WS; see Claim 6.*

### 7.2. Physics of Effective Temperatures

We have considered the RNC engine as a prototype of an engine with non-isothermal processes that require a *continuous* set of heat mediums of continuously varying temperatures between the two fixed temperatures $T_{0C}$ and $T_{0H}$, as discussed in Section 4.1. It is this part that introduces an effective working substance-dependent temperature so that they change as the working substance is changed. In contrast, $E^{\mathrm{RC}}$ and $E^{\mathrm{RC}\nu }$—see Conclusion 2—allow for only *fixed* isothermal processes and follow Protocol A so that their efficiencies are working substance-independent.

**Remark** **8.** *From the above discussion, we see that what is important for the performance of the engine are the two macroheats $\Delta_{e}Q_{C}$ and $\Delta_{e}Q_{H}$, having the ratio ρ in Equation (41) that eventually determines the ratio $T_{0C}^{*}/T_{0H}^{*}<1$ of the effective temperatures after entropy cancellation. Thus, we can consider a* hypothetical *reversible Carnot engine $E^{*\mathrm{RC}}$, working between the two effective temperatures $T_{0C}^{*}$ and $T_{0H}^{*}$, at which it rejects and accepts $\Delta_{e}Q_{C}$ and $\Delta_{e}Q_{H}$, respectively. Its efficiency $\epsilon^{*\mathrm{RC}}$ is precisely the* same *as $\epsilon^{\mathrm{RNC}}$ of $E^{\mathrm{RNC}}$. However, $\epsilon^{*\mathrm{RC}}$ is working substance-dependent due to $T_{0C}^{*}/T_{0H}^{*}$, which makes it very different from $\epsilon^{\mathrm{RC}}$, which is working substance-independent. This is the reason we use an asterisk to distinguish the two here.*

**Remark** **9.** *It should be emphasized that the effective temperatures are thermodynamically determined quantities by the response of WS, so they have as much physical significance as thermodynamic energy, macroheat, entropy, thermodynamic temperatures, etc., as discussed in Section 7.2. Thus, $E^{*\mathrm{RC}}$ and $E^{\mathrm{RNC}}$ are physically indistinguishable, even though they have different protocols. However, as $T_{0C}^{*}$ and $T_{0H}^{*}$ are determined thermodynamically, $E^{*\mathrm{RC}}$ has WS-dependent efficiency. It is common to use the following inequality; see Equation (49),*

(51) $$\epsilon^{\mathrm{RNC}}<\epsilon^{\mathrm{RC}}=1-\frac{T_{0C}}{T_{0H}},$$

*which, presumably appealing to some and certainly a valid inequality, does not have any physical significance as the efficiency of the hypothetical engine $E^{*\mathrm{RC}}$, from which we conclude that its motive power $\Delta_{e}W_{\mathrm{cyc}}=\epsilon^{*\mathrm{RC}}\Delta_{e}Q_{H}$. Any comparison with $\epsilon^{\mathrm{RC}}$ has no physical relevance whatsoever; it only results in a mathematical inequality with no physical significance as it conveys no information about WS dependence or which of the many $E^{\mathrm{RNC}}$s is* most efficient *or* least efficient*, which is where the physics lies.*

To further clarify the above remark, we see that knowing the efficiency of an engine is *upper-bounded* by the Carnot efficiency of a reversible Carnot engine, as shown in Equation (51), provides no information about the engine’s efficiency itself in that one *cannot* make any comparison between the efficiencies of different engines. On the other hand, identifying the fictitious Carnot engine with effective temperatures gives immediate information about its efficiency so that one can determine immediately which engine is most efficient. This is the benefit and usefulness of identifying the hypothetical engine $E^{*}$ associated with some engine E.

We now provide another viewpoint to show the physical relevance of effective temperatures that are defined thermodynamically. We will simplify the discussion and consider $E^{\mathrm{CR}2}$, but the discussion is easily generalized to any ν. Let us consider some $E^{\prime \mathrm{RC}}$ operating between the two temperatures $T_{0H}^{*}$ and $T_{0C}^{*}$, and ensure that its $Q_{\mathrm{acc}}^{\prime }=Q_{\mathrm{acc}}=Q_{1\mathrm{acc}}+Q_{2\mathrm{acc}}$ and $Q_{\mathrm{rej}}^{\prime }=Q_{\mathrm{rej}}=Q_{1\mathrm{rej}}+Q_{2\mathrm{rej}}$. Then, over the entire cycles of the two engines, they have $W^{\prime }=W$ and $Q_{\mathrm{acc}}^{\prime }=Q_{\mathrm{acc}}$. Thus, thermodynamically, there is no difference between the performances of the two engines even though they have different protocols.

### 7.3. ARC Engine $E^{\mathrm{ARC}}$ (Protocol B)

The discussion of effective temperatures above can now be applied to study $E^{\mathrm{ARC}}$, introduced in Section 4.2, in which the two isotherms in $E^{\mathrm{RC}}$ are replaced with the continuous sets of $\left\{\Sigma_{\mathrm{hH}}\left(V\right),\Sigma_{\mathrm{hC}}\left(V\right)\right\}$, with temperature sets $\left\{T_{0H}\left(V\right),T_{0C}\left(V\right)\right\}$ at each *V* of non-adiabatic processes $\left\{P_{\mathrm{acc}},P_{\mathrm{rej}}\right\}$ along ABC and CDA in Figure 4. Following the approach in determining $Q_{\mathrm{acc}4}$ in the previous subsection, we find

$$Q_{\mathrm{acc}}=\int_{\mathrm{ABC}}TdS\equiv T_{0H}^{*}\left(S_{C}-S_{A}\right)$$

along $P_{\mathrm{acc}}$, and

$$Q_{\mathrm{rej}}=\int_{\mathrm{CDA}}TdS\equiv T_{0C}^{*}\left(S_{A}-S_{C}\right)$$

along $P_{\mathrm{rej}}$. Using Equation (42), we immediately find

(52) $$\epsilon^{\mathrm{ARC}}=1-\frac{T_{0C}^{*}}{T_{0H}^{*}}<1,$$

which is again in the same form as $\epsilon^{\mathrm{RC}}$, but with effective temperatures $T_{0C}^{*}$ and $T_{0H}^{*}$ satisfying the temperature inequality in Equation (50). Again, there is entropy cancellation in the ratio ρ as before, but this fact has nothing to do with WS dependence in $\epsilon^{\mathrm{ARC}}$, as we are again dealing with Protocol B here.

We can now summarize the above conclusions in the form of the following:

**Claim** **8.** *It should be obvious from the use of the mean-value theorem of calculus that the general formulation of ϵ in Equation (40) of any arbitrary reversible cycle (RNC cycle or ARC cycle) requiring a continuous set of heat mediums results in*

(53) $$\epsilon =1-\frac{T_{0C}^{*}}{T_{0H}^{*}}<1,$$

*which looks similar in form to that of a* fictitious reversible Carnot engine *$E^{*\mathrm{RC}}$ of efficiency $\epsilon^{*\mathrm{RC}}=\epsilon^{\mathrm{ARC}}$; the values of $T_{0C}^{*}$ and $T_{0H}^{*}$ are working substance-independent—see Claim 7, Remarks 8 and 9, and Equation (8d)—but always satisfy the inequality in Equation (50). The physics behind the effective temperatures above still follows the discussion in Section 7.2.*

## 8. C-Th-2 and Its Consequences

### 8.1. Carnot’s Logical Proof

Carnot’s approach (reductio ad absurdum) to obtain his principle is a great example of the tour de force of using simple logic to obtain an unassailable foundational conclusion. It is just a method of proving that a premise is false since its logical consequence is absurdity. Carnot did not use any convoluted mathematical exploits to conclude C-Th-2. His proof that the reversible cycle has the maximum efficiency is reproduced below. However, we modify it slightly, but not in spirit, so that it can be extended to more complex classical heat engines and to quantum heat engines for our purpose.

Let us imagine that there is an *imaginary* (reversible or irreversible) engine EIm *undergoing the same four steps* (isotherms and adiabats) in and *following the same protocol* by the Carnot engine $E^{\mathrm{RC}}$ shown in Figure 2, but may be executing them somewhat differently, such as at different speeds.

**Remark** **10.** *The phrases in italics are our addition, which are not part of C-Th-2, but will be important in our analysis to extend C-Th-2.*

**Remark** **11.** *The italic phrases put a restriction on the two engines. For example, let EIm represent the reversible engine $E^{\mathrm{RC}2}$ discussed in Section 5. This engine requires two additional heat mediums over and above the two mediums that are going to be shared by $E^{\mathrm{RC}}$. Even though they both follow Fix-Prtocol A, they have a different number of steps. Therefore, there is no way to connect them after reversing $E^{\mathrm{RC}}$ to satisfy the condition of no effect on the heat mediums that is central to the proof as we now show.*

Accordingly, we need to consider the modified C-Th-2M in Section 2.2, which clearly specifies the restriction to only two isotherms. Therefore, we will demand EIm to operate between the same two isotherms and using the same protocol as in $E^{\mathrm{RC}}$ so that they can be compared together. Observe that no such restriction is mentioned in C-Th-2 by Carnot himself, and this has created some confusion in the literature and had to be clarified [17]. We will *not* allow EIm to represent the reversible engine $E^{\mathrm{RC}2}$ as noted above as they have different protocols. In order to compare the two engines, we assume that EIm has higher efficiency

(54) ϵIm>ϵRC

than the reversible Carnot engine $E^{\mathrm{RC}}$, with both working between the same but distinct heat mediums that are classified by their temperatures, and that both accept the *same* amount of exchange macroheat $\Delta_{e}Q_{H}$ from the outside and reject the same amount $\Delta_{e}Q_{C}$ to the outside. Thus, different efficiencies mean that

ΔeWIm>ΔeWRC.

We now reverse $E^{\mathrm{RC}}$, which we denote by ${\bar{E}}^{\mathrm{RC}}$ and use EIm to drive reversed ${\bar{E}}^{\mathrm{RC}}$ so that the latter accepts $\Delta_{e}{\bar{Q}}_{\mathrm{acc}}\equiv \Delta_{e}Q_{C}$ from and returns $\Delta_{e}{\bar{Q}}_{\mathrm{rej}}\equiv \Delta_{e}Q_{H}$ to the outside so that each heat medium remains *unaffected*, which plays a very important role in the Clausius logic. The combined engine $E^{\mathrm{ImRC}}≐$EIm∪E¯RC is able to create useful macrowork

ΔeWImRC≐ΔeWIm−ΔeWRC>0

without having any impact on the outside. Thus, we have created a *perpetual-motion* machine that can be used to generate an infinite amount of macrowork from nowhere. Such a possibility of a perpetual-motion machine is an impossibility (something he learned about from his father, Lazare [15]) so the prior assumption in Equation (54) must be false.

Thus, there are only two possibilities:

1. ϵIm≡ϵRC. In this case, there cannot be any perpetual-motion machine. Both EIm and $E^{\mathrm{RC}}$ must be reversible Carnot machines. This means that *all* reversible engines *undergoing the same four steps* have the same efficiency, which can also be written as $\epsilon_{\mathrm{rev}}$ to reflect that its entire operation is based on the same reversible processes.
2. ϵIm>ϵRC. In this case, EIm must be any irreversible engine *undergoing the same four steps*, but which must involve dissipation due to irreversibility, so its efficiency, which we denote by $\epsilon_{\mathrm{irr}}$, reflects the effect of this dissipation. Thus,
  (55) $$\epsilon_{\mathrm{irr}}<\epsilon_{\mathrm{rev}}.$$

**Remark** **12.** *We emphasize that the logical proof offered by Carnot makes no statement about protocols of the engine, and it being a classical engine or a quantum engine [25,26] (even though quantum mechanics was not even invented in his time) so both kinds of engines must satisfy the conclusion in Equation (55). The proof also does not require knowing the absolute temperatures as they were yet to be identified at his time. We have also not used the first or the second law (law of increase in entropy) so whether Carnot believed at this point in the caloric theory is also irrelevant for the proof. However, it has become evident from Carnot’s fragment notes that he did not believe in the caloric theory afterwards; see his quote in Reflections-1.*

As Carnot was only considering engines that were working between the same two fixed isotherms, the restriction imposed by the extra phrases above—see Remark 10—does not have any effect as both engines perform the same set of operations (processes) using the same protocol. However, these phrases will be important below.

### 8.2. Extension to Other Engines

We stress that we have only required the additional phrase—see Remark 10—in the above proof. Thus, the proof can be equally applied to any reversible engines that differ from $E^{\mathrm{RC}}$ in the specification of any or all of the four of its processes and any protocols. Once the processes and protocols are specified for reversible engines such as for $E^{\mathrm{RN}\nu },E^{\mathrm{RNC}},E^{\mathrm{ARC}}$, etc., we can replace $E^{\mathrm{RC}}$ in the above proof by Carnot and compare its efficiency with that of any imaginary EIm undergoing the same identical processes. If we do, we will come to the same conclusion as the two possibilities listed above. Thus, if we conclude that Equation (55) is satisfied, then the imaginary engine corresponding to $E^{\mathrm{RNC}},E^{\mathrm{ARC}}$, etc. must be their irreversible versions, respectively.

We now see the importance of including the phrase in Remark 10 in C-Th-2. Engines must be properly classified by the nature of processes involved in their performance. This allows us to now make a stronger statement related to C-Th-2M and C-Th-3M as follows, by adding another letter E for extension, as follows:

**Theorem** **1.** C-Th-2ME*: All heat engines operating under a* prescribed *protocol set of processes (which may differ from the set of processes in the RC engine $E^{\mathrm{RC}}$) and that accept and reject macroheats at higher and lower (effective) temperatures, respectively, cannot have efficiency greater than its reversible analogs.*

Different reversible engines performing over a prescribed cycle $P_{\mathrm{cyc}}$ differ in WS employed in the engine. We now provide the extension of C-Th-3M to reversible heat engines different from the Carnot engine $E^{\mathrm{RC}}$, as follows:

**Theorem** **2.** C-Th-3ME*: The efficiency of a reversible heat engine operating under a* prescribed *protocol set of processes specified by $P_{\mathrm{cyc}}$ that accepts and rejects macroheats at several higher and lower temperatures, respectively, and that* differs *from the protocol set of processes in the RC engine $E^{\mathrm{RC}}$ is equal to that of a (fictitious) Carnot engine $E^{*\mathrm{RC}}$, which accepts and rejects macroheats at higher and lower (effective) temperatures, respectively, as given in Equations (35) and (53).*

The importance of the above extensions is that they identify the exact value of the efficiency $\epsilon^{R}$ of some engine $E^{R}$, while C-Th-2M only provides an upper bound to it by $\epsilon^{\mathrm{RC}}$, but not its exact value. Recall that $E^{\mathrm{RC}}$ and $\epsilon^{\mathrm{RC}}$ carry no information of prescribed cycle and protocol of $E^{R}$ and whose efficiency $\epsilon^{R}$ is given in terms of effective cold and hot temperatures in Equation (53) in Claim 8.

An important aspect of the extension is that it allows us to determine which engine has higher efficiency, which is not possible from knowing the upper bound. Thus, the upper bound is surely not useful for comparative investigation.

The incorporation of protocols is also very important. We turn to Fermi’s Corollary 1 of the Carnot theorem, which only deals with efficiency. We consider two reversible engines $E^{\mathrm{RC}}$ and $E^{\mathrm{RNC}}$. According to the corollary, they should have the same efficiency, which is obviously incorrect. Thus, protocols become very important to remove this inconsistency. This ensures that the only *reversible engine* with *two and only two* isothermal heat mediums is the reversible Carnot engine; all *other* engines $E^{R}$ with two and only two variable heat mediums or with more than two isothermal or variable heat mediums can be cast as a fictitious Carnot Engine $E^{*\mathrm{RC}}$ and efficiency $\epsilon^{*\mathrm{RC}}$ with effective temperatures, as noted in C-Th-2M. The extension says that the efficiency of any irreversible engine $E^{\mathrm{Ir}}$ cannot exceed $\epsilon^{*\mathrm{RC}}$. As the reversible efficiency $\epsilon^{*\mathrm{RC}}$ is known, the extension allows us to determine the exact loss of efficiency of $E^{\mathrm{Ir}}$ relative to $\epsilon^{*\mathrm{RC}}$. This is a much stronger result than the claim that the weak upper bound $\epsilon^{\mathrm{Ir}}\leq \epsilon^{\mathrm{RC}}$.

**Remark** **13.** *Carnot made no assumptions about the nature of WS used in the two engines EIm and $E^{\mathrm{RC}}$ in his proof, as the latter is working substance-independent; see Claim 7 and Remark 8. However, these other reversible engines differ from $E^{\mathrm{RC}}$ in that the efficiencies of the former may be working substance-dependent.*

As any arbitrary reversible ARC cycle can be decomposed into a large number of RC cycle, as discussed in Section 4.2, we focus on this decomposition. As $\epsilon_{j}^{\mathrm{RC}}$ is maximum for $E_{j}^{\mathrm{RC}}$, the efficiency $\epsilon^{\mathrm{ARC}}$ of the ARC engine $E^{\mathrm{ARC}}$ is also the maximum for the prescribed set of processes and WSs; see its bound in Equation (31). This is also the efficiency of the fictitious Carnot engine $E^{*\mathrm{RC}}$; see Theorem 2. Any irreversible analog of $E^{\mathrm{ARC}}$ for the same set of processes and WS has an efficiency that cannot exceed $\epsilon^{\mathrm{ARC}}$.

This now completes the discussion of all reversible engines.

## 9. Thermodynamic Inconsistency of Regenerators: Stirling Engine

### 9.1. Reversible Regenerator with Any WS

The topic of regenerators is not central to our investigation, but is included here as there may be some criticism of our determination in Section 7.1 of the efficiency $\epsilon^{\mathrm{RNC}}$ of the engine $E^{\mathrm{RNC}}$ that follows the cycle in Figure 3 by bringing in the concept of regeneration [16] (see Section 10.6.5). The issue appears for the reversible Stirling engine, Otto engine, and Ericsson engine, but here we will only focus on the Stirling engine $E^{S}$ following the cycle in Figure 3. The two isotherms at $T_{0H}$ ($P_{1}$) and at $T_{0C}$ ($P_{3}$) still remain as part of the cycle. The isochoric processes ($P_{2}$ and $P_{4}$) of $E^{\mathrm{RNC}}$ introduced in Section 4.1 are used in the Stirling engine [17,18,21], where they replace the two adiabats in $E^{\mathrm{RC}}$. Therefore, to treat $E^{S}$ as a *reversible* engine $E^{\mathrm{RS}}$, we need to carry out these processes in the presence of an infinite sequence of mediums [16] (see 5.14.1 and 10.6.5) with temperatures ranging between $T_{0C}$ and $T_{0H}$ as before. As these temperatures are determined by the WS of $E^{\mathrm{RS}}$ (Protocol B), its efficiency $\epsilon^{\mathrm{RS}}$ will depend on it as follows from our discussion of $E^{\mathrm{RNC}}$. We mostly follow Kestin, who has derived many of the results presented in this section.

Engineers and scientists have used the regenerative cycle [16] (see p. 184) to get around this issue for $E^{\mathrm{RS}}$ by introducing flows through the regenerator and to argue that it has the same efficiency as $E^{\mathrm{RC}}$ [21] (see p. 92) as we further elaborate in Section 9.2. This also requires this version of the Stirling engine to be *reversible*. As Kestin [16] (see p. 498) notes, adiabats of $E^{\mathrm{RC}}$ are replaced by

“⋯ different reversible processes during which elements of heat dQ are exchanged at all intermediate processes. Such a cycle is sketched in Figure 10.18(a). ⋯ between any isotherm *T* and the neighboring isotherm T+dT⋯”

Thus, reversibility in $E^{\mathrm{RS}}$ demands a continuous distribution of heat mediums between $T_{0C}$ and $T_{0H}$ as noted above, and before for $E^{\mathrm{RNC}}$.

Indeed, Atkins specifically mentions that “ … the cycle is gone through quasistatically, …” [21] (see p. 92) without specifying how to accomplish this; also no restriction on the WS is made in the discussion by Atkins. In view of these assumptions by Atkins, and following C-Th-3 or C-Th-3M, the implication is that the efficiency must be independent of WS. This is a very surprising result, which directly *contradicts* the discussion of $\epsilon^{\mathrm{RNC}}$ in Section 7.1. This casts serious doubts on the conclusion by Atkins; however, see Section 9.2 for details.

Unfortunately, the above conclusion by Atkins works for $E^{\mathrm{RS}}$ *if and only if* it uses the ideal gas as the WS but not for other substances, a fact Atkins fails to mention. Indeed, one must make sweeping approximations [27,28] (for example) to justify Atkins’s above claim. This means that there are *conflicting views* of what a Stirling engine $E^{S}$ with regenerator is. Indeed, as Salter [18] notes, the confusion started with not properly understanding a reversible regenerator; see also Liley [29].

To understand the above critique, we assume that the regenerator operates *reversibly* as assumed by Atkins to ensure that the engine is reversible. We follow Equations (44a) and (44b) for quasistatic processes, according to which, $E^{\mathrm{RS}}$ rejects macroheat

(56a) $$Q_{\mathrm{rej}}^{\mathrm{reg}}=\int_{2\to 3}C_{V}\left(T,V_{2}\right)dT,$$

as $T_{0H}\to T_{0C}$ along $P_{2}$, and accepts macroheat

(56b) $$Q_{\mathrm{acc}}^{\mathrm{reg}}=\int_{4\to 1}C_{V}\left(T,V_{1}\right)dT,$$

as $T_{0C}\to T_{0H}$ along $P_{4}$. Even though $T_{0C}$ and $T_{0H}$ are fixed so the range of integration is the same, the two macroheats have in general different magnitudes as $C_{V}\left(T,V\right)$ changes with *V*. Thus, there remains a net balance from the two macroheats in that

(57) $$Q_{\mathrm{acc}}^{\mathrm{reg}}+Q_{\mathrm{rej}}^{\mathrm{reg}}\neq 0.$$

This imbalance destroys one of the conditions [16] (see p. 501) of a thermodynamically consistent regenerative cycle. Incidentally, this also destroys the assumption by Atkins that WS “… is heated by the energy previously stored …” in the regenerator, which requires an equality in Equation (57). The other condition about the entropy change [16] (see 5.14.1) is also violated, as we now demonstrate. The corresponding entropy changes

(58a) $$\Delta S_{\mathrm{rej}}^{\mathrm{reg}}=\int_{2\to 3}C_{V}\left(T,V_{2}\right)dT/T$$

(58b) $$\Delta S_{\mathrm{acc}}^{\mathrm{reg}}=\int_{4\to 1}C_{V}\left(T,V_{1}\right)dT/T$$

also have different magnitudes for the same reason so that

(59) $$\Delta S_{\mathrm{acc}}^{\mathrm{reg}}+\Delta S_{\mathrm{rej}}^{\mathrm{reg}}\neq 0.$$

As the two conditions discussed by Kestin [16] (see pp. 498 and 501) are violated, it shows that the regenerator concept applied to $E^{\mathrm{RS}}$ generally leads to *thermodynamic inconsistency* so one cannot justify that the Stirling engine has the same efficiency equal to $\epsilon^{\mathrm{RC}}$, as discussed by Atkins [21], Barkat [30], and others [17,18] (for example).

**Conclusion** **4.** *The fundamental condition of a reversible Carnot engine $E^{\mathrm{RC}}$ is given in Equation (20), which is re-expressed in Equations (25b) and (42). However, because of the inequality in Equation (59), the cyclic property $\Delta S_{\mathrm{cyc}}=0$ in Equation (9) is also* violated *if $E^{\mathrm{RS}}$ is treated similar to a Carnot engine $E^{\mathrm{RC}}$ that must satisfy Equation (22). This immediately disproves any connection between $E^{\mathrm{RS}}$ and $E^{\mathrm{RC}}$, as is suggested above by Atkins and others.*

Indeed, Salter makes a very confusing statement that “… the cycle is a pair of adiabatic stages linked by isothermal stages, which means that the Stirling cycle with a reversible regenerator is just a different way to implement a Carnot cycle”. Even if we consider the equality in Equation (59) for ideal gas, it is *incorrect* to claim that each isochore is an adiabat. This is evident from Figure 1(b) in [18], where the isochores are not vertical at fixed *S*. It appears that the statement is only made to justify Atkins’s claim, which we have just shown in Conclusion 4 to be invalid. Indeed, we establish in Section 9.2 that isochores are not adiabats; see Equations (60) and (66).

**Remark** **14.** *As entropy is an integral part of the thermodynamics of any engine, the inequality in Equation (59) immediately requires that $Q_{\mathrm{acc}}^{\mathrm{reg}}$ in the Stirling engine cannot be* neglected*, and must include ($Q_{\mathrm{acc}4}≐Q_{\mathrm{acc}}^{\mathrm{reg}}$ ) to determine $Q_{\mathrm{acc}}$—see Equation (45)—as is conducted in determining the efficiency of $E^{\mathrm{RNC}}$, as is conducted in Section 7.1.*

Seldman and Mlchalik [17] use our definition of efficiency in Equation (2); see also Salter [18].

### 9.2. Heat Exchanger Irreversibility and Ideal Gas

The ideal gas has been singled out above for the simple reason that the inequalities in Equations (57) and (59) turn into equalities so that

(60) $$Q_{\mathrm{acc}}^{\mathrm{reg}}=-Q_{\mathrm{rej}}^{\mathrm{reg}},\Delta S_{\mathrm{acc}}^{\mathrm{reg}}=-\Delta S_{\mathrm{rej}}^{\mathrm{reg}}.$$

This means that macroheats along the two isochores cancel out, leaving behind only $Q_{\mathrm{acc}1}$ along 1→2 and $Q_{\mathrm{rej}3}$ along 3→4, so

(61) $$Q_{\mathrm{cyc}}=Q_{\mathrm{acc}1}+Q_{\mathrm{rej}3},$$

as if $Q_{\mathrm{acc}}^{\mathrm{reg}}$ and $Q_{\mathrm{rej}}^{\mathrm{reg}}$ do not “exist;” recall that all these macroheats are exchange macroheats since *Q* refers to $\Delta_{e}Q$. This is an interesting observation and has been interpreted by engine theorists [21,30] (for example) to treat $Q_{\mathrm{acc}1}$ as $Q_{\mathrm{acc}}$ in Equation (2), and not to consider the original definition

(62) $$Q_{\mathrm{acc}}=Q_{\mathrm{acc}1}+Q_{\mathrm{acc}}^{\mathrm{reg}},Q_{\mathrm{rej}}=Q_{\mathrm{rej}3}+Q_{\mathrm{rej}}^{\mathrm{reg}}$$

along $P_{\mathrm{acc}}$ and $P_{\mathrm{rej}}$, see Definition 1, that was followed in the original calculation in Equation (45). The efficiency in Equation (2) using Equation (45) gives

(63) $$\epsilon^{\mathrm{RS}}≐\epsilon^{\mathrm{RNC}}<\epsilon^{\mathrm{RC}},$$

a result that is valid not just for ideal gas but for any WS. As Protocol B is used, $\epsilon^{\mathrm{RS}}$ depends on the WS. Kestin [16] (see Equation (10.56)) also uses the above Carnot efficiency.

Instead, by not including $Q_{\mathrm{acc}}^{\mathrm{reg}}$ in $Q_{\mathrm{acc}}$ so that we follow the substitution in Equation (3), we identify the non-Carnot efficiency $\epsilon_{\mathrm{NC}}^{\mathrm{RS}}$ in Equation (4)

(64) $$\epsilon_{\mathrm{NC}}^{\mathrm{RS}}≐\frac{W}{Q_{\mathrm{acc}1}}\equiv \frac{Q_{\mathrm{acc}}}{Q_{\mathrm{acc}1}}\epsilon^{\mathrm{RNC}}\geq \epsilon^{\mathrm{RNC}}$$

for $E^{\mathrm{RS}}$ and for any WS, as also obtained by Kestin [16] (see Equations (10.56a) and below it), who calls it the regeneration efficiency.

However, for ideal gas, we have $\Delta S_{\mathrm{acc}}^{\mathrm{reg}}=-\Delta S_{\mathrm{rej}}^{\mathrm{reg}}$ from Equation (60), a condition that is imposed by Kestin [16] (see Equation (10.57a)) for a reversible regenerator, so

$$\Delta S_{\mathrm{cyc}}=\Delta S\left(P_{1}\right)+\Delta S\left(P_{3}\right)=0;$$

we also have

$$Q_{\mathrm{acc}1}=T_{0H}\Delta S\left(P_{1}\right),Q_{\mathrm{rej}3}=T_{0C}\Delta S\left(P_{3}\right),$$

which allows for simplifying the result for $\epsilon_{\mathrm{NC}}^{\mathrm{RS}}$:

(65) $$\epsilon_{\mathrm{NC}}^{\mathrm{RS}}=1-Q_{\mathrm{rej}3}/Q_{\mathrm{acc}1}=\epsilon^{\mathrm{RC}},\;(\mathrm{Ideal}\;\mathrm{Gas}),$$

another remarkable result often found in the literature [21,30] (for example) but not with much clarification. The exception is Kestin [16] (see Equations (10.57a)). We have now justified that the result holds only for $E^{\mathrm{RS}}$ using reversible generator and ideal gas.

The above result has encouraged engineers to take a bold step: *replace* the reversible regenerator in Section 9.1 with a *heat exchanger* (hexc), whose role is to reject $Q_{\mathrm{rej}}^{\mathrm{hexc}}$ by cooling the ideal gas from $T_{0H}$ to $T_{0H}$ along $P_{2}$, store it, and then use this stored macroheat to heat the ideal gas from $T_{0C}$ to $T_{0H}$ along $P_{4}$. This is a major change in the protocol for $E^{\mathrm{RS}}$, so we identify the resulting engine as *modified* Stirling engine $E_{\mathrm{mod}}^{S}$ for a reason that will become clear below. One can use a *fixed* and *finite* number of more than one heat exchanger kept at different temperatures, but we will focus on a single one here for simplicity.

The discussion about whether $E_{\mathrm{mod}}^{S}$ is a reversible engine remains very confusing in the literature. An example of such a discussion is by Barakat et al. [30] (p. 4, Section 2.1), where the following is stated:

In the regenerator, the heat released during isochoric cooling is balanced by that absorbed during isochoric heating, and this heat exchange is reversible. Therefore, external heat transfer exclusively takes place during isothermal expansion and compression.

In addition, Barakat et al. [30] (p. 4, Section 2.1) also state that one of the *assumptions* that is made is the following:

There are no internal losses in the engine, including leakage, friction, and mechanical losses.

It is not the consumption of stored macroheat that determines reversibility or irreversibility; rather, it is the production of entropy (irreversible entropy generation) that determines whether the process is reversible or not. We remind the reader of Figure 1b in [18] and Figure 10.18(a) in Kestin [16], which show changes in the entropy during the two processes, but say nothing about entropy generation.

The above assumption in [30] (p. 4, Section 2.1) suggests that the modified Stirling engine must be treated as reversible. That will require an infinite heat medium, as was considered in Section 9.1, and by Kestin [16]. Unfortunately, this assumption is factually incorrect for heat exchangers in general, as documented by various workers who have provided detailed calculations of irreversible entropy generation; see, for example [31,32,33].

Here, we will assume for simplicity of computation the following modified protocol.

**Remark** **15.** Modified Protocol *: In this protocol, the heat exchanger as regenerator is maintained at fixed temperatures $T_{0C}$ during $P_{2}$ and $T_{0H}$ during $P_{4}$.*

We do not discuss the mechanism ensuring fixed volume and macroheat storage. It is easy to extend the discussion below when the two ends of the heat exchanger are at fixed temperatures $T_{0C}$ and $T_{0H}$ instead of the above protocol without affecting the content of Theorem 3. We now justify the following:

**Conclusion** **5.** *$E_{\mathrm{mod}}^{S}$ must be identified as an* irreversible *engine $E_{\mathrm{mod}}^{\mathrm{IrS}}$. Additionally, because of this irreversibility with the heat exchanger, there is no reversible analog of $E_{\mathrm{mod}}^{\mathrm{IrS}}$. Most importantly, it must not be confused with the reversible engine $E^{\mathrm{RS}}$.*

Let us focus on entropy changes $\Delta S_{\mathrm{rej}}^{\mathrm{hexc}}\left(P_{2}\right)$ and $\Delta S_{\mathrm{acc}}^{\mathrm{hexc}}\left(P_{4}\right)$ during $P^{\mathrm{hexc}}≐P_{2}\cup P_{4}$. They are equal and opposite, being equal to the entropy differences between two equilibrium macrostates, and thus they cancel out. As

$$\Delta S=\Delta_{e}S+\Delta_{i}S,$$

we have the net change over $P^{\mathrm{hexc}}$

(66) $$\Delta S^{\mathrm{hexc}}=\frac{Q_{\mathrm{rej}}^{\mathrm{hexc}}}{T_{0C}}+\frac{Q_{\mathrm{acc}}^{\mathrm{hexc}}}{T_{0H}}+\Delta_{i}S^{\mathrm{hexc}}=0,$$

where $Q_{\mathrm{rej}}^{\mathrm{hexc}}/T_{0C}$ and $Q_{\mathrm{acc}}^{\mathrm{hexc}}/T_{0H}$ denote the exchange entropy changes $\Delta_{e}S^{\mathrm{hexc}}$ over $P^{\mathrm{hexc}}$; recall from Equation (60) that

(67) $$Q_{\mathrm{acc}}^{\mathrm{hexc}}=-Q_{\mathrm{rej}}^{\mathrm{hexc}},$$

and that there is no irreversibility along $P_{1}$ and $P_{3}$. We now prove the following

**Theorem** **3.** *The entropy generation in the heat exchanger is non-negative ($Q_{\mathrm{acc}}^{\mathrm{hexc}}=-Q_{\mathrm{rej}}^{\mathrm{hexc}}=0$), making $E_{\mathrm{mod}}^{S}$* irreversible, *so we write it as $E_{\mathrm{mod}}^{\mathrm{IrS}}$.*

**Proof.**  We use Equation (67) in Equation (66) to express

(68) $$\Delta_{i}S^{\mathrm{hexc}}=Q_{\mathrm{acc}}^{\mathrm{hexc}}\left(1/T_{0C}-1/T_{0H}\right)>0.$$

as $T_{0C}<T_{0H}$. This proves the theorem. □

To determine the entropy change over the entire modified cycle, we need to recall $\Delta_{i}S=\Delta S^{\mathrm{hexc}}$ over the entire cycle so that

(69) $$\Delta S_{\mathrm{cyc}}=Q_{\mathrm{acc}}/T_{0H}+Q_{\mathrm{rej}}/T_{0C}+\Delta_{i}S=0,$$

where we have used total macroheats $Q_{\mathrm{acc}}≐Q_{\mathrm{acc}1}+Q_{\mathrm{acc}}^{\mathrm{hexc}}$ and $Q_{\mathrm{rej}}≐Q_{\mathrm{rej}3}+Q_{\mathrm{rej}}^{\mathrm{hexc}}$ to express the entropy change $\Delta S_{\mathrm{cyc}}$; observe that we cannot overlook $Q_{\mathrm{acc}}^{\mathrm{hexc}}$ and $Q_{\mathrm{rej}}^{\mathrm{hexc}}$ in this calculation, as they appear with different coefficients, as seen from Equation (66). This immediately justifies Definition 1 to use $Q_{\mathrm{acc}}$ and $Q_{\mathrm{rej}}$, as we have conducted in Section 7.1 of the efficiency $\epsilon^{\mathrm{RNC}}$ and to use the Carnot efficiency in Equation (2).

We now determine the efficiency of $E_{\mathrm{mod}}^{\mathrm{IrS}}$ by following Gujrati [34] (see Section 12), where irreversibility has been included in the discussion. We use $W=Q_{\mathrm{acc}}+Q_{\mathrm{rej}}$ for the engine, which is identical to Equation (61), and use it in Equation (69) to finally obtain

(70) $$W/T_{0C}=Q_{\mathrm{acc}}\left(1/T_{0C}-1/T_{0H}\right)-\Delta_{i}S^{\mathrm{hexc}},$$

which includes a negative contribution form $\Delta_{i}S=\Delta_{i}S^{\mathrm{hexc}}$. This is identical to the result given in Equation (136a) in [34] (see Section 12, and Equation (137)), where *W* is reduced by irreversibility due to non-isothermal processes. We use *W* above to finally obtain

(71) $$\epsilon_{\mathrm{mod}}^{\mathrm{IrS}}=W/Q_{\mathrm{acc}}\equiv \epsilon^{\mathrm{RC}}-T_{0C}\Delta_{i}S^{\mathrm{hexc}}/Q_{\mathrm{acc}},\;(\mathrm{Ideal}\;\mathrm{Gas}),$$

a result given in [34] (see Equation (137)) with $\Delta_{i}S=\Delta_{i}S^{\mathrm{hexc}}$; compare with Equation (65).

We emphasize that the presence of $\epsilon^{\mathrm{RC}}$ in Equation (71) obtained by using Definition 1 for ideal gas should not give the false hope that $\epsilon_{\mathrm{mod}}^{\mathrm{IrS}}=\epsilon^{\mathrm{RC}}$ is the efficiency of some reversible version of $E_{\mathrm{mod}}^{\mathrm{RS}}$ for any WS. Indeed, the above conclusion is only valid for ideal gas so it is not very interesting to compare this efficiency with that of the reversible Carnot engine, where any WS is allowed. It is easy to see that the conclusion does not change if we finitely consider many heat exchangers, each with a fixed temperature, or the one with its two ends that are maintained at fixed $T_{0C}$ and $T_{0H}$, respectively.

There seems to be a tendency in the literature [16,17,18,21] to use the non-Carnot efficiency in Equation (3) by not following Definition 1. Using Equation (3), we find that

(72) $$\epsilon_{\mathrm{mod},\mathrm{NC}}^{\mathrm{IrS}}≐W/Q_{\mathrm{acc}1}=\left(Q_{\mathrm{acc}}/Q_{\mathrm{acc}1}\right)\epsilon_{\mathrm{mod}}^{\mathrm{IrS}}>\epsilon_{\mathrm{mod}}^{\mathrm{IrS}},$$

where $Q_{\mathrm{acc}1}=Q_{\mathrm{acc}1}\left(P_{1}\right)$ and $Q_{\mathrm{acc}}/Q_{\mathrm{acc}1}=1+Q_{\mathrm{acc}}^{\mathrm{hexc}}\left(P_{4}\right)/Q_{\mathrm{acc}1}$. This should not be a surprise, as $\epsilon_{\mathrm{mod},\mathrm{NC}}^{\mathrm{IrS}}$ is obtained by dividing by a smaller amount of heat. However, there is a direct and simple way to determine $\epsilon_{\mathrm{mod},\mathrm{NC}}^{\mathrm{IrS}}$ as follows. We express Equation (69) differently as follows:

$$\Delta S_{\mathrm{cyc}}=Q_{\mathrm{acc}1}/T_{0H}+Q_{\mathrm{rej}3}/T_{0C}=0.$$

Using $W=Q_{\mathrm{acc}1}+Q_{\mathrm{rej}3}$, and following the above steps to derive Equation (70), we obtain

$$W/T_{0C}=Q_{\mathrm{acc}1}\left(1/T_{0C}-1/T_{0H}\right)$$

so that we finally obtain the non-Carnot efficiency

(73) $$\epsilon_{\mathrm{mod},\mathrm{NC}}^{\mathrm{IrS}}≐W/Q_{\mathrm{acc}1}=\epsilon^{\mathrm{RC}},\;(\mathrm{Ideal}\;\mathrm{Gas}),$$

which is equal to the Carnot efficiency $\epsilon^{\mathrm{RC}}$ of $E^{\mathrm{RC}}$ usually ascribed to a Stirling engine with heat exchanger and using ideal gas; it is indeed higher than the Carnot efficiency $\epsilon^{\mathrm{RC}}$ of the reversible Carnot engine $E^{\mathrm{RC}}$.

**Conclusion** **6.** *Let us compare various efficiencies of the Stirling engine $E^{S}$ using ideal gas; thus, we obtain the following values:*

(74) $$\begin{array}{c} \epsilon^{\mathrm{RS}}=\epsilon^{\mathrm{RNC}}<\epsilon^{\mathrm{RC}},\epsilon_{\mathrm{mod}}^{\mathrm{IrS}}<\epsilon^{\mathrm{RC}}\\ \epsilon_{\mathrm{NC}}^{\mathrm{RS}}=\epsilon^{\mathrm{RC}},\epsilon_{\mathrm{mod},\mathrm{NC}}^{\mathrm{IrS}}=\epsilon^{\mathrm{RC}}, \end{array}$$

*with the top row giving Carnot efficiencies and the bottom row giving non-Carnot efficiencies, for reversible and irreversible Stirling engines, respectively. It is interesting to compare $\epsilon^{\mathrm{RS}}$ with $\epsilon_{\mathrm{NC}}^{\mathrm{RS}}$. Their respective values follow the inequality in Equation (3), which is expected as they refer to different measures of efficiency. However, a surprising and troubling aspect is revealed when we compare $\epsilon_{\mathrm{mod}}^{\mathrm{IrS}}$ with $\epsilon_{\mathrm{mod},\mathrm{NC}}^{\mathrm{IrS}}$. While their magnitudes follow the inequality in Equation (3), as expected, $\epsilon_{\mathrm{mod}}^{\mathrm{IrS}}$ reveals the irreversibility aspect of $E_{\mathrm{mod}}^{\mathrm{IrS}}$ as seen from the presence of $\Delta_{i}S^{\mathrm{hexc}}$ in Equation (71), but this contribution is missing in $\epsilon_{\mathrm{mod},\mathrm{NC}}^{\mathrm{IrS}}$, in which the irreversible segments of $P_{\mathrm{cyc}}$ have been discarded. This issue is taken up in Section 9.3. We do not compare $\epsilon^{\mathrm{RS}}$ with $\epsilon_{\mathrm{mod}}^{\mathrm{IrS}}$, as protocols for $E^{\mathrm{RS}}$ and $E_{\mathrm{mod}}^{\mathrm{IrS}}$ are* different*. Thus, we do not treat $E^{\mathrm{RS}}$ and $E_{\mathrm{mod}}^{\mathrm{IrS}}$ together to ensure that $E^{\mathrm{RS}}$ is not confused as the reversible analog of $E_{\mathrm{mod}}^{\mathrm{IrS}}$. This conclusion is consistent with Conclusion 5.*

The above conclusion summarizes the results for the Stirling engine with ideal gas. Thus,

**Conclusion** **7.** *We have established that the Carnot efficiency of the modified Stirling engine $E_{\mathrm{mod}}^{\mathrm{IrS}}$ with ideal gas is less than the Carnot efficiency $\epsilon^{\mathrm{RC}}$ of the reversible Carnot engine due to irreversibility, while the non-Carnot efficiency of $E_{\mathrm{mod}}^{\mathrm{IrS}}$ equals $\epsilon^{\mathrm{RC}}$. Thus, the non-Carnot efficiency is not capable of capturing irreversibility due to segments that are not considered in the non-Carnot efficiency. Because of this, the non-Carnot efficiency must not be taken as a thermodynamic measure of the efficiency of a heat engine unless we wish to* demolish the Carnot theorem*, which is a cornerstone of classical thermodynamics by not capturing the irreversibility inherent in the engine.*

### 9.3. Dissipation and Irreversibility

We now provide an explanation of the lack of dissipation in Equation (73), which requires appreciating the subtle difference between (work) dissipation and irreversibility. Irreversibility can be due to work dissipation and due to irreversible heat flow at different temperatures. Therefore, to determine work dissipation, i.e., lost work, we need to subtract the irreversible entropy generation due to heat flow from the total entropy generation. We have discussed this point in the literature at various places, but most recently in [35], where Equation (30) gives dissipation after the subtraction. In the case of the heat exchanger along isochores, there is no dissipation as the macrowork is identically zero. The irreversible entropy generation $\Delta_{i}S=\Delta_{i}S^{\mathrm{hexc}}$ is only due to irreversible heat flow, which is consistent with no work dissipation. This explains the mystery behind Equation (73). This also explains the price of overlooking irreversible entropy generation from the heat exchanger in determining the non-Carnot efficiency. Thus, as said above, this efficiency must not be taken as a thermodynamic measure of efficiency that is captured by the Carnot theorem.

## 10. Discussion and Summary

We now turn to the main topics of this study on the technical aspect of the contribution that deals with the Carnot engine, Carnot theorem, and their various extensions. We mainly discuss, what we consider to be, new results, to the best of my knowledge.

### 10.1. Generalization of Carnot’s Theorem

We have carefully looked at the Carnot theorem—see its three parts C-Th-1, C-Th-2, and C-Th-3—in his own words in Section 2 and the renditions of the last two in the modern literature by C-Th-2 and C-Th-3M, given in Section 2.2.

We noted the miraculous cancellation of entropy differences—see Equation (5)—in the ratio ρ in Equation (41) that reduced it to $T_{0C}/T_{0H}$. This has resulted in making Carnot’s reversible engine $E^{\mathrm{RC}}$ very special among *all* possible reversible heat engines. What is special is that it is the only one whose efficiency is determined by the ratio $T_{0C}/T_{0H}$ that is fixed by the external heat mediums (Protocol A) so it becomes independent of its working substance; see Conclusion 3 and Remark 7, which clearly show the important contributions Carnot made to thermodynamics. The efficiencies of all other reversible engines can be cast into a form similar to that of $\epsilon^{\mathrm{RC}}$ but in terms of effective temperatures—see Conclusion 2, Claims 6–8—so these efficiencies may or may not depend on the WS. Therefore, all other reversible engines must be identified, not only by its processes, but also by its working substance, as discussed in Section 2.3 and Section 2.4 to uniquely specify engines. Given the two, all of their irreversible analogs must have their efficiencies not exceed that of the reversible engine, as noted in Section 8. However, knowing $\varepsilon^{\mathrm{RC}}$ as the loose upper bound for the efficiency does not help determine whether a certain engine is more efficient than another. Here comes the usefulness of the effective temperatures so that all reversible engines can be identified with a hypothetical reversible Carnot engine $E^{*\mathrm{RC}}$ working between its two effective temperatures that are process (protocol) and may be substance-dependent. Knowing the efficiency $\epsilon^{*\mathrm{RC}}$ of the hypothetical engine $E^{*\mathrm{RC}}$ allows for a direct comparison of the effectiveness of various engines. The importance of protocols contained in these results is summarized in the form of Theorems 1 and 2, that extend C-Th-2M and C-Th-3M, given in Section 2.2, and provide an extension of Carnot’s theorem (C-Th-2 and C-Th-3) to other reversible engines different from Carnot’s engine $E^{R}$. This finally provides the justification of one of the most important new results that were stated as Conclusion 1 in Section 1.3. The irreversibility of an engine of a particular cycle (protocol) is due to following the cycle irreversibly. Thus, we must compare this irreversible engine with a reversible analog having the same protocol. Comparing an irreversible engine using one protocol with a reversible engine with a different protocol can result in violating the Carnot theorem, as expressed in Conclusions 5 and 6. As the Carnot theorem is the foundation of classical thermodynamics, we must ensure that it is never violated. This is why we need to modify the Carnot theorem. These two conclusions show the importance of protocols, without which these engines will not be covered by the Carnot theorem. Protocols also play an important role in extending Carnot’s logical proof in Section 8.

We stated the three parts of the famous Carnot theorem (C-Th-1,C-Th-2,C-Th-3) in Section 1.1 and their modern expressions (C-Th-2M, C-Th-3M). The main conclusions were announced under 1, 2, and 3 in Section 1.2, which we have now expanded into Theorems 1 and 2 in Section 8.

However, we should again mention Conclusions 5 and 6, which refer to the case where an irreversible engine has no reversible analog. This happens when a segment of the cycle is designed as irreversible such as the one using a heat exchanger with a fixed temperature along its walls or between its two ends; as the WS flows through the exchanger, it undergoes irreversible entropy generation as seen in Theorem 3.

### 10.2. Role of the Working Substance

We introduced for the first time in the heat engine literature two different classes of protocols, Protocol A and Protocol B, to distinguish those engines whose efficiencies are WS-independent and those whose efficiencies are WS-dependent. We clarify this interplay by invoking a thermodynamic argument that the interplay forces the macrostates of the engine to adjust to fit the protocol. To the best of our knowledge, such an explanation is put forward for the first time.

### 10.3. Carnot’s Approach

We show in Section 3.1 how Carnot could estimate the efficiency of his reversible engine even without having the first law at his disposal and using a now-discredited caloric theory. It is indeed surprising that even with this handicap, he obtained a profound result about the relationship between the efficiency $\epsilon^{\mathrm{RC}}$ of $E^{\mathrm{RC}}$ that he never obtained in a closed form, and other engines whose efficiency can never exceed this. The last result was a demonstration of his skillful manipulation of logic, which is discussed in Section 8.

There is some misconception about this last result (C-Th-3M). According to it, all reversible engines operating *between* the same two heat mediums are equally efficient. Could one have other heat mediums apart from the two that lie between these two? This point requires clarification [17], so we introduced a reversible engine $E^{\mathrm{RC}2}$ formed by two $E^{\mathrm{RC}}$s connected in parallel in Section 5, and demonstrated that it does not satisfy C-Th-3M. The operational details are most certainly different. Its efficiency is lower than $\epsilon^{\mathrm{RC}}$, which is usually interpreted as that $E^{\mathrm{RC}2}$ must represent an irreversible engine. Even this is false, as $E^{\mathrm{RC}2}$ is reversible.

This has required us to impose further conditions on comparing efficiencies, which is reflected in Remarks 10 and 11 for a sensible comparison of engines of any kind. Such limitations have never been discussed in the literature.

### 10.4. First Law and S  from Carnot’s Viewpoint

We have attempted to provide a conjectural discussion of how one can deduce the first law for a cycle by what was known to Carnot before his untimely death, and simple dimensional analysis is presented in Section 3. We have acknowledged that Carnot had abandoned the caloric theory and had recognized the mechanical equivalence between *W* and $Q_{\mathrm{acc}}$, as noted in Equation (1) and discussed further in Section 3.2. The first law for a cycle appears in Equation (21) and the cycle efficiency appears in Equation (23). We introduced the notion of a ratio ρ in Equation (15) and a new quantity *R* so that its change ΔR, called the *Carnot ratio* and defined in Equation (24), plays an important role that gave the clue to Clausius [2] to introduce his entropy *S* since

ΔS≡ΔR

in all cases. Because of this, we recommend that *S* should be called *Carnot–Clausius entropy*.

### 10.5. Calorique and Entropy

As Carnot had abandoned the caloric theory after the publication of *Reflections*, there is really no need to ever wonder if he used caloric as entropy, even though many have and still do [36], to equate the two with a goal to clarify some misconceptions that have arisen form Carnot’s writing or to build on the caloric theory. We have discussed this issue immediately before Equation (1) and again in Section 3.4, with a different perspective. Our connection between caloric and entropy is via the Carnot ratio ΔR. For us, they are related but not identical via the Clausius equality dQ=TdS, according to which *S* is a state function, but there is no state function corresponding to dQ. In other words, dS is not a process quantity, but dQ is.

### 10.6. Stirling Engine with Reversible/Irreversible Regenerator

As we discussed in Section 9 and revisit here, the issue of reversible regeneration and, in particular, its irreversible replacement by one or finite many heat exchangers, has given rise to many unsubstantiated claims that cannot be logically justified. The confusion is caused by two distinct objectionable claims:

1. Heat exchangers must be treated as internal components of the engine $E^{S}$ so exchanged macroheats with them must be internal and reversible.
2. Ideal gas as the WS ensures that Equation (67) must be satisfied so they can be ignored so as to allow using Equation (65), which violates Definition 1, to determine efficiency.

Both claims are not supported by thermodynamics.

Regarding point 1, we observe that it seems to be limited to ideal gas as the WS, so this situation is not relevant to the Carnot theorem that is the focus of this study. However, because of many misconceptions in this regard, we have decided to look at this case for its relevance to the limitation of the original Carnot theorem. Considering the simple model of the heat exchanger we have employed, its temperature is fixed at $T_{0C}$ over $P_{2}$ for WS to cool down from $T_{0H}$. Thus, the heat exchanger must be in thermal contact with the colder medium used during $P_{3}$ so the heat exchanger is not truly internal. Similarly, it must be in thermal contact with the hotter medium used during $P_{1}$ so it is again not truly internal during $P_{4}$. In both processes, it must be treated as an *extension of the external heat mediums* so that it remains in equilibrium with them. However, this fact is not relevant as far as the stored heat is concerned, which is assumed to be completely transferred internally. However, the irreversibility is associated, not with the stored heat transfer, but with the entropy change in the WS, as shown in Equation (66). The situation does not change if we modify the above simple model so that the two ends of the heat exchanger are maintained at $T_{0C}$ and $T_{0H}$; see [31,32,33]. The irreversible entropy generation makes the modified Stirling engine *irreversible*. Despite this, its Carnot (and not the non-Carnot) efficiency is given in Equation (71), which includes the contribution from irreversibility in the irreversible engine. However, the presence of $\epsilon^{\mathrm{RC}}$ must not give the reader the impression that $\epsilon^{\mathrm{RC}}$ represents the efficiency of its reversible analog engine “$E_{\mathrm{mod}}^{\mathrm{RS}}$” of $E_{\mathrm{mod}}^{\mathrm{IrS}}$. This is not correct, as $E_{\mathrm{mod}}^{\mathrm{IrS}}$ is truly irreversible, as Theorem 3 establishes. Unless we change the protocol, the heat exchanger cannot be turned into a reversible heat exchanger.

However, we find that the non-Carnot efficiency $\epsilon_{\mathrm{mod},\mathrm{NC}}^{\mathrm{IrS}}$ in Equation (73) shows no effect of irreversibility inherent in $E_{\mathrm{mod}}^{\mathrm{IrS}}$. The remarkable result is explained in Section 9.3, where we discuss the difference between work dissipation and irreversible entropy generation only due to macroheat exchange at different temperature. In the presence of irreversibility only due to macroheat exchange, there cannot be any work dissipation [35]. This is the explanation of the above remarkable result in Equation (73). The efficiency $\epsilon^{\mathrm{RS}}$ of the reversible Stirling engine $E^{\mathrm{RS}}$ is given in Equation (63) and is most certainly strictly upper bounded by $\epsilon^{\mathrm{RC}}$. This clearly shows that $\epsilon^{\mathrm{RC}}$ in Equation (71) should not be confused with the efficiency of the true reversible Stirling engine $\epsilon^{\mathrm{RS}}$ by assuming that we allow walls of the heat exchanger to have its temperature vary continuously between $T_{0C}$ and $T_{0H}$. It we make this incorrect identification, we have an irreversible engine $E_{\mathrm{mod}}^{\mathrm{IrS}}$, whose efficiency $\epsilon_{\mathrm{mod},\mathrm{NC}}^{\mathrm{IrS}}$ is higher than that of its (incorrect) reversible analog $E^{\mathrm{RS}}$, which is a violation of the Carnot engine. Thus, $E^{\mathrm{RS}}$ must *not* be considered the reversible analog of $E_{\mathrm{mod}}^{\mathrm{IrS}}$; see Conclusion 6.

It is interesting to consider the non-Carnot efficiency $\epsilon_{\mathrm{NC}}^{\mathrm{RS}}$ by overlooking the macroheats along $P_{2}$ and $P_{4}$ for ideal gas, which is given in Equation (65). Let us contrast it with $\epsilon_{\mathrm{mod},\mathrm{NC}}^{\mathrm{IrS}}=\epsilon^{\mathrm{RC}}$, as given in Equation (72), of the irreversible engine $E_{\mathrm{mod}}^{\mathrm{IrS}}$. A complete tabulation of various efficiencies is given in Equation (74). Now, we can make the heat exchanger reversible by letting its temperature vary continuously between $T_{0C}$ and $T_{0H}$ as above so $\epsilon_{\mathrm{mod},\mathrm{NC}}^{\mathrm{IrS}}$ turns into $\epsilon_{\mathrm{mod},\mathrm{NC}}^{\mathrm{RS}}$. This is again problematic for the Carnot theorem for the same reason as above: irreversibility is not captured by $\epsilon_{\mathrm{mod},\mathrm{NC}}^{\mathrm{IrS}}$. This shows that the non-Carnot efficiency does not fit the Carnot theorem Corollary 1 by Fermi so it does not represent a thermodynamic measure of efficiency; see also Conclusions 5 and 6. Our final conclusion is captured in Conclusion 7.

We are thankful to various referees for useful criticism of the manuscript that has helped in improving it. We take the responsibility for all shortcomings.

## Data Availability

The original contributions presented in this study are included in the article. Further inquiries can be directed to the corresponding author.

## List of Acronyms and Notation

acc	AcceptedMacroheat
ARC	Arbitrary Reverse Cycle
C-Th-*i*	Carnot Theorem-*i*, *i* = 1,2,3
C-Th-*i*M	Modern Carnot Theorem-*i*, *i* = 2,3
C-Th-*i*ME	Extension of C-Th-*i*M, *i* = 2,3
$d_{e}Q,Q\;=\;\Delta_{e}Q$	Exchange Macroheat
$d_{e}W,W\;=\;\Delta_{e}W$	Exchange Macrowork
Fix	Fixed (Protocol)
E,ϵ	Any Engine, Efficiency
$E^{*},\epsilon^{*}$	Fictitious Engine, Efficiency
$E^{C},\epsilon^{C}$	Carnot Engine, Efficiency
EIm,ϵIm	Imaginary Engine, Efficiency
$E^{\mathrm{Ir}},\epsilon^{\mathrm{Ir}}$	Irreversible Engine, Efficiency
$E^{\mathrm{IrC}},\epsilon^{\mathrm{IrC}}$	Irreversible Carnot Engine, Efficiency
$E^{\mathrm{IrS}},\epsilon^{\mathrm{IrS}}$	Irreversible Stirling Engine, Efficiency
$E^{R},\epsilon^{R}$	Reversible Engine, Efficiency
$E^{\mathrm{RC}},\epsilon^{\mathrm{RC}}$	Reversible Carnot Engine, Efficiency
$E^{\mathrm{RC}2\;\mathrm{or}\;\nu },\epsilon^{\mathrm{RC}2\;\mathrm{or}\;\nu }$	Two or ν $E^{\mathrm{RC}\prime }$s in parallel, Efficiency
$E^{\mathrm{RNC}},\epsilon^{\mathrm{RNC}}$	Reversible Non-Carnot Engine, Efficiency
$E^{\mathrm{RS}},\epsilon^{\mathrm{RS}}$	Reversible Stirling Engine, Efficiency
$E^{S},\epsilon^{S}$	Stirling Engine, Efficiency
$E_{\mathrm{mod}}^{S},\epsilon_{\mathrm{mod}}^{S}$	Modified Stirling Engine, Efficiency
$\epsilon_{\mathrm{rev},\mathrm{irr}\;}$	Efficiency: Reversible, Irreversible Engine
$\epsilon_{\min,\max\;}^{\mathrm{RC}}$	Minimum, Maximum Efficiency of $E_{j}^{\mathrm{RC}}$
Fix	Fix Protocol A
hexc	Heat Exchanger
NonFix	NonFix Protocol B
NC	Non-Carnot
rej	Rejected Macroheat
WS	Working Substance
